# Engineered nanovesicle platform simultaneously triggers YAP-dependent ferroptosis and reprograms T-cell immunity through miR-150-3p codelivery in melanoma microenvironment

**DOI:** 10.7150/thno.115860

**Published:** 2025-07-25

**Authors:** Jiemin Wang, Zhenguo Zhao, Haopeng Yang, Ruixuan Wang, Shu Wang, Jiale Yu, Yujia Wang, Ruihua Liu, Yani Chen, Yueshi Liu, Kesong Shi, Pengyong Han, Miao Liu, Jing Miao, Xiaoyang Li, Xiangnan Li, Haiquan Yu

**Affiliations:** 1 State Key Laboratory of Reproductive Regulation and Breeding of Grassland Livestock, School of Life Sciences, Inner Mongolia University, Hohhot 010020, Inner Mongolia, China.; 2 Department of Orthopaedics, National Cancer Center/National Clinical Research Center for Cancer/Cancer Hospital, Chinese Academy of Medical Sciences and Peking Union Medical College, 100021, Beijing, China.

**Keywords:** extracellular vesicles, miR-150-3p, ferroptosis, tumor microenvironment, hippo-YAP signaling

## Abstract

**Rationale:** Melanoma remains a highly aggressive malignancy with limited effective therapies and frequent resistance to immune checkpoint blockade (ICB). Extracellular vesicles (EVs) represent a promising platform for RNA-based therapeutics, but their clinical translation is impeded by inefficient cargo loading and insufficient tumor-specific targeting. To address these limitations, we developed an engineered EV strategy integrating efficient miRNA packaging with tumor-targeting surface modifications to enhance therapeutic outcomes in melanoma.

**Methods:** Engineered EVs (iEV-150) were generated by co-expressing miR-150-3p and Annexin A2 (ANXA2) in HEK293T cells, followed by surface modification with tumor-targeting iRGD peptides. Mechanistic insights were obtained using RNA sequencing, RNA immunoprecipitation (RIP), chromatin immunoprecipitation (ChIP), and luciferase reporter assays. Ferroptosis induction was evaluated through lipid peroxidation analysis, mitochondrial membrane potential assays, and transmission electron microscopy (TEM). Therapeutic efficacy and biodistribution were assessed *in vivo* using subcutaneous and metastatic melanoma mouse models. Immune modulation was examined by analyzing CD8⁺ T cell activation via flow cytometry in co-cultures of patient-derived CD8⁺ T cells and melanoma cells treated with iEV-150.

**Results:** miR-150-3p was elevated in melanoma-derived EVs, and ANXA2 was identified as a key RNA-binding protein that selectively facilitated its loading into EVs. iEV-150 exhibited enhanced uptake by melanoma cells and improved tumor-specific accumulation *in vivo*. Mechanistically, iEV-150 suppressed NF2 expression, disrupted the NF2-LATS1 interaction, activated YAP signaling, and subsequently upregulated ferroptosis-related genes ACSL4 and CHAC1, thereby inducing ferroptosis through the NF2-Hippo-YAP axis. In addition to its direct anti-tumor effects, iEV-150 promoted CD8⁺ T cell infiltration and activation within the tumor microenvironment, and significantly enhanced the therapeutic efficacy of ICB in melanoma models.

**Conclusions:** iEV-150 integrates ANXA2-mediated miRNA loading, tumor-specific targeting, ferroptosis induction, and immune microenvironment reprogramming. This engineered EV strategy provides an effective RNA-based therapeutic platform to overcome ICB resistance and enhance precision treatment in melanoma.

## Introduction

Extracellular vesicles have emerged as promising nanocarriers for RNA-based cancer therapy because of their intrinsic biocompatibility, efficient cargo protection, and inherent ability to mediate intercellular communication 1, 2. Compared with synthetic nanocarriers, engineered EVs exhibit superior potential for targeted therapy, offering increased efficacy and reduced off-target effects 3-5. Despite these advantages, clinical translation remains hindered by challenges such as low cargo-loading efficiency and insufficient tumor-specific targeting, emphasizing the need for further optimization of EV-based precision therapies 6-8.

In melanoma, tumor-derived EVs substantially contribute to disease progression by remodeling the TME. These vesicles facilitate immune evasion, angiogenesis, and metastasis, primarily through the transfer of bioactive molecules, notably microRNAs (miRNAs) 9-12. This EV-mediated crosstalk often fosters an immunosuppressive TME, undermining the effectiveness of immunotherapies such as ICB 13.

Recent advances in EV engineering, particularly the use of peptide-functionalized EVs, offer promising solutions to these challenges. For example, EVs conjugated with ischemic myocardium-targeting peptides successfully delivered miR-30d to damaged cardiac tissues, mitigating cardiac hypertrophy 14. Similarly, EVs modified with integrin α5-targeting peptides effectively reprogrammed cancer-associated fibroblasts (CAFs) in pancreatic cancer, thereby remodeling the TME 15. These studies highlight the potential of engineered EVs for precise RNA delivery and their prospective utility in melanoma therapy. Tumor-derived EVs naturally serve as carriers for miRNAs, shielding them from enzymatic degradation and enhancing their cellular uptake 16.

Specific miRNAs enriched in EVs can profoundly influence tumor progression 17, 18. Among these, miR-150-3p, a tumor-suppressive miRNA, has potent inhibitory effects on melanoma, partly by targeting critical oncogenes such as KLF4 19. In addition to its role in melanoma, miR-150-3p has been shown to have antitumor effects on head and neck squamous cell carcinoma 20, colorectal cancer 21, and lung adenocarcinoma 22. Despite its therapeutic potential, the clinical application of miR-150-3p is limited by its instability, poor intracellular delivery, and lack of tumor-specific targeting. Thus, a reliable and precise delivery platform for miR-150-3p is urgently needed.

Ferroptosis, a recently described iron-dependent form of cell death characterized by lipid peroxidation and reactive oxygen species (ROS) accumulation, has emerged as a new therapeutic target 23-26. Regulators of ferroptosis are increasingly recognized as potential avenues for cancer therapy. Glutathione-specific gamma-glutamylcyclotransferase 1 (CHAC1), a glutathione (GSH)-degrading enzyme, can decrease intracellular antioxidant defenses and increase ROS levels, thereby increasing ferroptosis sensitivity 27, 28. CHAC1 expression, which is regulated by the PERK-eIF2α-ATF4 pathway, is significantly upregulated by ferroptosis inducers such as erastin and RSL3, establishing CHAC1 as a critical biomarker of ferroptosis 29. Acyl-CoA synthetase long-chain family member 4 (ACSL4) is a long-chain fatty acid metabolic enzyme that facilitates the activation of polyunsaturated fatty acids (PUFAs) and their incorporation into phospholipids, thereby increasing cellular susceptibility to lipid peroxidation 30, 31. As a central ferroptosis regulator, ACSL4 is highly expressed in ferroptosis-prone cells, influencing tumor cell susceptibility to ferroptosis 32. Importantly, dysregulation of iron metabolism and aberrant expression of ferroptosis-related genes are significantly correlated with melanoma prognosis, highlighting the therapeutic potential of targeting ferroptosis pathways in melanoma treatment 33, 34. These findings collectively suggest that inducing ferroptosis and modulating iron homeostasis may offer therapeutic avenues for melanoma therapy.

**Scheme 1:**
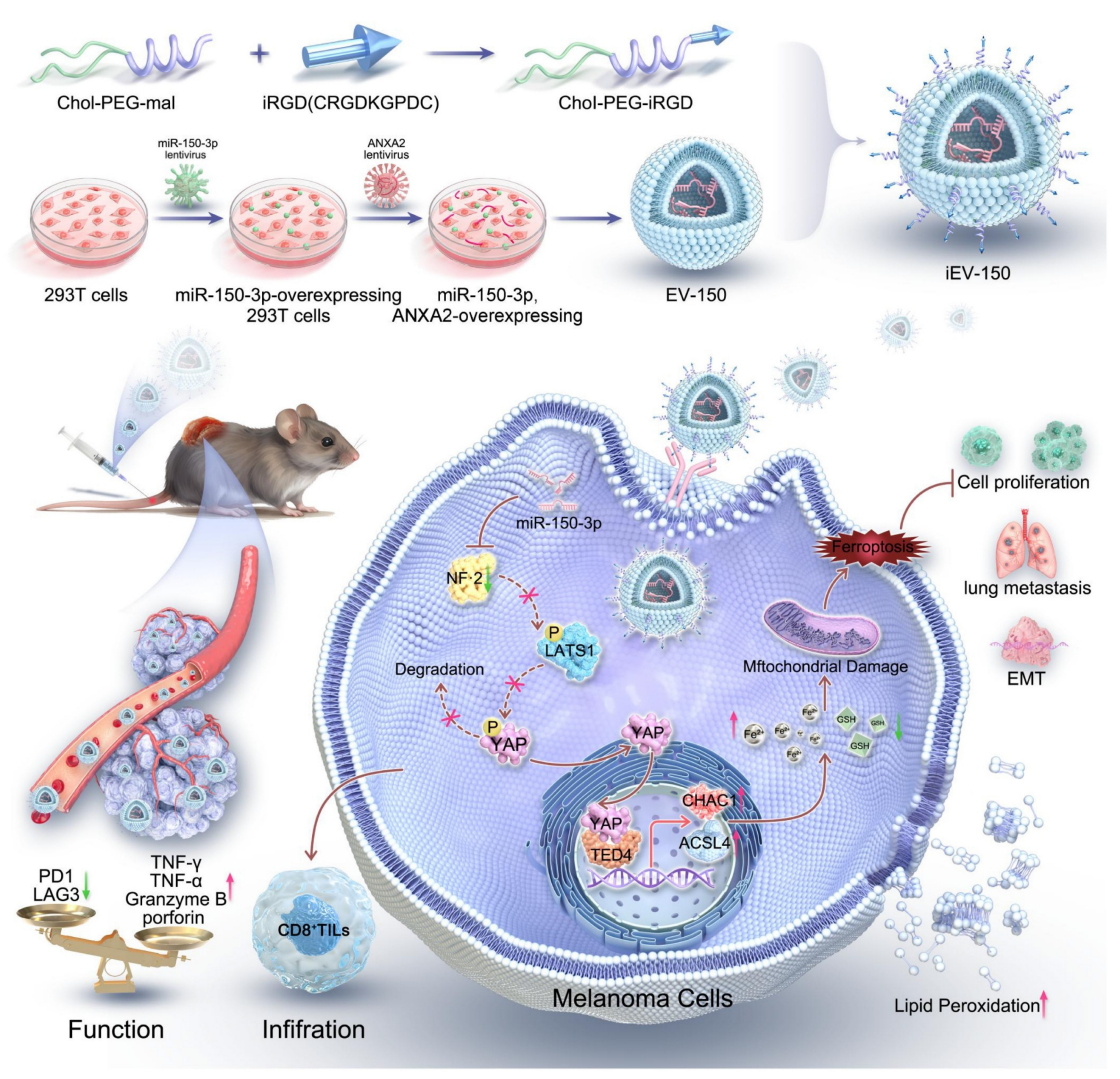
The engineered EVs platform by conjugating EVs with the tumor-homing peptide iRGD encapsulated with miR-150-3p, utilizing annexin A2 (ANXA2) to enhance selective RNA incorporation.

On the basis of these insights, an engineered EV (iEV-150) that displays the tumor-homing peptide iRGD on its surface was developed and loaded with miR-150-3p, utilizing annexin A2 (ANXA2) to enhance selective RNA incorporation. This strategy aims to achieve precise and efficient melanoma therapy. We investigated its mechanism of action, focusing on iEV-150-induced ferroptosis via the NF2-Hippo-YAP signaling axis and its effects on antitumor immunity in melanoma** (Scheme 1)**. Our findings established a robust framework for RNA-based EV therapeutics and highlight the transformative potential of engineered EVs in overcoming melanoma progression and therapeutic resistance.

## Results

### miR-150-3p is enriched in EVs secreted by melanoma, and blocking its transfer inhibits tumor cell proliferation

**Figure 1:**
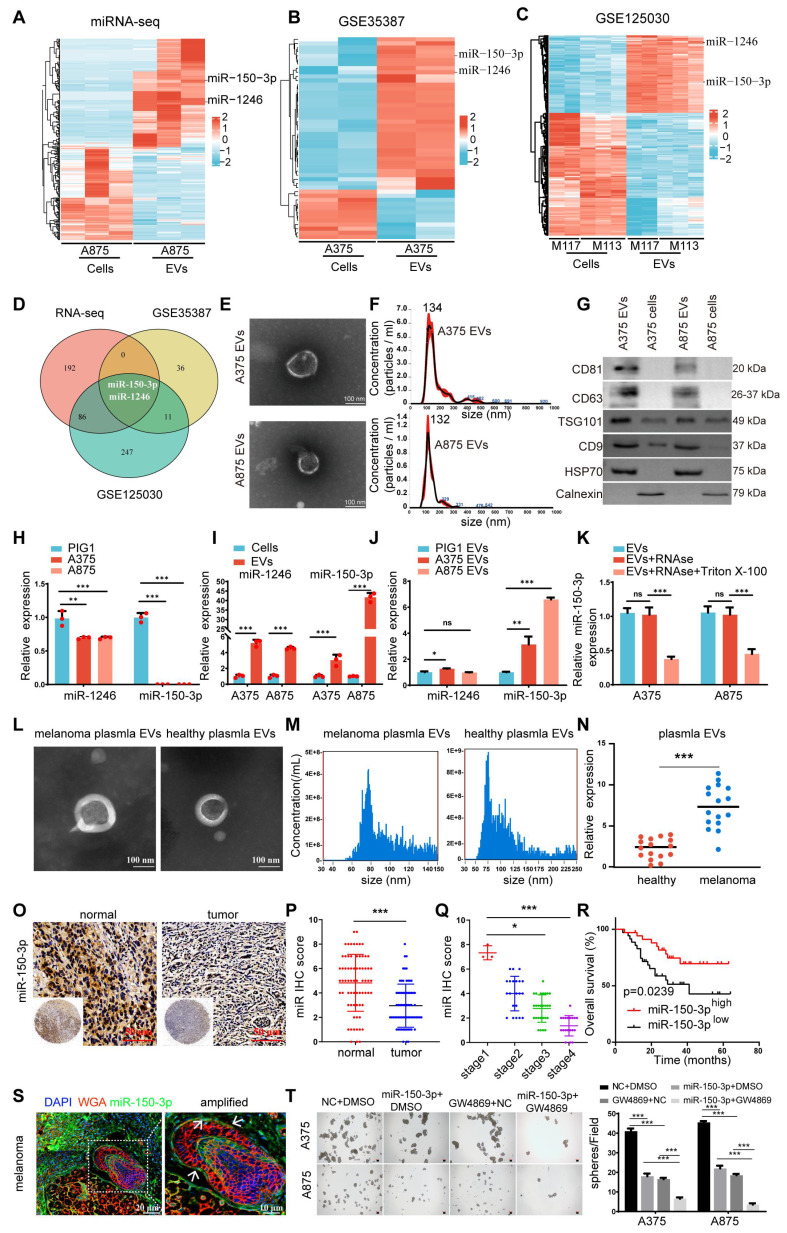
** miR-150-3p is enriched in EVs secreted by melanoma, and blocking its transfer inhibits tumor cell proliferation. A-C** Heatmaps showing the miRNA profiles of melanoma cells and their EVs.** D** Venn diagram. **E** TEM images of melanoma-derived EVs (scale: 100 nm). **F** NTA of EV size and concentration.** G** Western blot showing EV markers (CD9, CD63, CD81, TSG101, and HSP70) and a negative marker (calnexin). **H-J** qRT‒PCR analysis of miR-150-3p and miR-1246 in PIG1 cells vs. melanoma cells (H), melanoma cells vs. their EVs (I), and melanoma vs. normal cell-derived EVs (J). **K** miR-150-3p levels in EVs treated with RNase A and/or Triton X-100. **L** TEM of plasma-derived EVs from melanoma patients (scale: 100 nm). **M** Nanoflow cytometry analysis of the plasma EV size distribution. **N** qRT‒PCR of miR-150-3p in plasma EVs from melanoma patients and healthy donors. **O, P** ISH staining (O) and scoring (P) of miR-150-3p in melanoma vs. adjacent normal tissues (n = 70). Scale: 50 μm. **Q** ISH scores of miR-150-3p in tumor tissues from patients with clinical stage 1 (n = 3), stage 2 (n = 21), stage 3 (n = 27), or stage 4 (n =19) disease. **R** Kaplan‒Meier survival analysis of 70 melanoma patients stratified by high (n = 35) or low (n = 35) miR-150-3p expression.** S** Immunofluorescence detection of miR-150-3p in melanoma tissues (n = 70); WGA (red, membrane), DAPI (blue, nuclei), and miR-150-3p (green probe). Scale bar: 20 μm. **T** Spheroid formation assay of melanoma cells.

We conducted miRNA sequencing to analyze the differential expression of miRNAs between A875 melanoma cells and their EVs to identify miRNAs with key biological roles in melanoma (Figure 1A). A total of 135 downregulated and 143 upregulated miRNAs were identified in the A875 EVs (p < 0.05, |log_2_FC| > 2) (Figure S1A). In addition, we queried the gene expression database (GEO) for datasets related to melanoma cells and their derived EVs and reanalyzed the GSE35387 dataset (A375 cells vs. EVs) (Figure 1B) and the GSE125030 dataset (primary cells vs. EVs) (Figure 1C). By intersecting the results from these three datasets, we identified that miR-150-3p and miR-1246 were consistently upregulated in melanoma-derived EVs (Figure 1D). In A375 EVs, the levels of both miRNAs exceeded those in parental cells and normal control EVs (Figure S1B). We proceeded to isolate and characterize EVs from both A375 and A875 cells using transmission electron microscopy (Figure 1E), size distribution analysis (Figure 1F), and identification of specific EV markers (Figure 1G).

As shown in Figure 1H, miR-1246 and miR-150-3p were significantly downregulated in normal melanocytes (PIG1) compared with the tested melanoma cell lines. Conversely, these miRNAs were markedly upregulated in melanoma cell-derived EVs compared with the parental melanoma cells themselves (Figure 1I). Additionally, the level of miR-150-3p was significantly greater in melanoma cell-secreted EVs than in PIG1-derived EVs, whereas the level of miR-1246 was only modestly greater (Figure 1J). The expression of the extracellular vesicle miR-150-3p did not change after RNaseA treatment but decreased after both RNaseA and Triton X-100 treatment, suggesting that miR-150-3p was encapsulated (Figure 1K).

Furthermore, compared with those from healthy controls, the plasma EVs from 16 melanoma patients exhibited elevated miR-150-3p levels (Figure 1L-N). *In situ* hybridization (ISH) of melanoma tissue microarrays revealed lower miR-150-3p scores in tumor tissues than in adjacent normal tissues (Figure 1O-Q), with scores decreasing from stage 1 to stages 2-4 (p < 0.05; Figure 1Q). Consistently, Kaplan‒Meier analysis revealed that lower miR-150-3p ISH scores were associated with shorter overall survival (Figure 1R). Interestingly, miR-150-3p expression in melanoma tissues was observed not only in the cytoplasm but also as distinct aggregated signals in the extracellular space. These findings suggest that miR-150-3p may be incorporated into EVs, enabling them to exert biological effects within the melanoma microenvironment (Figure 1S).

To further explore the role of miR-150-3p in melanoma, we used GW4869, a specific inhibitor of nSMase that reduces sphingomyelin degradation on the membrane and decreases EV production. The inhibition of nSMase2 significantly reduced EV secretion by both A375 and A875 cells (Figure S1C), leading to a marked increase in the intracellular miR-150-3p level (Figure S1D). In stable miR-150-3p-overexpressing A375/A875 cells treated with GW4869 for 24 h, spheroid formation (Figure 1T), CCK-8 assays (Figure S1E), and cell cycle analysis (Figure S1F) demonstrated suppressed proliferation, while Western blot analysis confirmed reduced expression of vimentin, N-cadherin, snail, PCNA, cyclin E1, and CDK2, alongside elevated E-cadherin and P21 levels (Figure S1G). These findings suggest that the inhibition of EVs containing miR-150-3p leads to its accumulation within melanoma cells, thereby inhibiting cell proliferation and EMT.

### ANXA2 regulates the loading of miR-150-3p into EVs

Although EVs exhibit miRNA profiles distinct from those of their donor cells, the mechanisms driving this divergence remain elusive. Given the established role of RNA-binding proteins (RBPs) in sorting miRNAs into EVs, we aimed to identify RBPs associated with miRNAs enriched in cancer-derived EVs. To this end, we isolated biotin-labeled miR-150-3p complexes from A875 cells using streptavidin-coated magnetic beads. Sodium dodecyl sulfate‒polyacrylamide gel electrophoresis (SDS‒PAGE) revealed a characteristic staining pattern for these Bio-miR-150-3p complexes (Figure S2A). Proteins uniquely present in the Bio-miR-150-3p complexes but absent in the control Bio-miRNA complexes were identified via in-gel digestion and liquid chromatography‒mass spectrometry (LC‒MS) analysis (Table S1). Subsequent GO and KEGG enrichment analyses of these proteins revealed ANXA2, RAB14, TMED9, VAPA, and ARF4 as key regulators of EV transport pathways (Figure S2B).

To validate these interactions, we performed RNA immunoprecipitation (RIP) assays targeting the RBPs highlighted by the GO and KEGG analyses. IgG served as a negative control to rule out nonspecific binding, with an anti-GAPDH antibody used as a housekeeping control. Notably, miR-150-3p levels in ANXA2 immunoprecipitates were more than 13-fold higher than those in IgG controls, underscoring a robust and specific interaction (Figure S2C). In contrast, the TMED9 immunoprecipitates showed moderate enrichment of miR-150-3p, whereas the levels in the RAB14, VAPA, and ARF4 immunoprecipitates were comparable to those in the IgG baseline (Figure S2C). We further confirmed the interaction between miR-150-3p and ANXA2 by RNA pull-down assays, with Western blot analysis used as the readout (Figure S2D). To ascertain whether the interaction involved direct binding, recombinant GST-ANXA2, purified from *E. coli*, was incubated with biotin-labeled miR-150-3p and captured using streptavidin-coated beads. Western blot analysis verified that ANXA2 directly binds to miR-150-3p (Figure S2E).

Consistent with these findings, electrophoretic mobility shift assays (EMSAs) revealed supershifts when miR-150-3p was incubated with ANXA2 (Figure S2F). Immunofluorescence analysis further revealed the colocalization of miR-150-3p and ANXA2 in melanoma cells (Figure S2G). To explore the functional role of ANXA2, we overexpressed ANXA2 in melanoma cells (Figure S2H, bottom panel). While this did not increase EV release (Figure S2I), it significantly reduced the intracellular miR-150-3p level (Figure S2J) and markedly increased its enrichment in melanoma-derived EVs (Figure S2K). Conversely, CRISPR-mediated ANXA2 knockout (KO) in melanoma cells, achieved using ANXA2 sgRNA lentivirus transduction, substantially reduced ANXA2 expression (Figure S2H, top panel; Figure S2L, left panel), leading to elevated intracellular miR-150-3p levels (Figure S2L, left panel). This increase was inversely correlated with diminished miR-150-3p levels in secreted EVs (Figure S2L, right panel and Figure S3A). These findings collectively indicate that ANXA2 mediates the sorting of miR-150-3p into EVs.

We next assessed the impact of ANXA2 KO on melanoma cell proliferation using a CCK-8 assay, which revealed significant suppression of A375 and A875 cells (Figure S2M and Figure S3B). This finding aligns with the reduced protein levels of PCNA and CCND1 (Figure S2N). Notably, inhibiting miR-150-3p expression reversed the suppressive effects of ANXA2 KO on PCNA and CCND1 expression, restoring cell proliferation (Figure S2M-N). In parallel, we evaluated ANXA2 expression in melanoma. qRT‒PCR analysis revealed increased ANXA2 mRNA levels in melanoma cell lines compared with normal melanocytes (Figure S3C), whereas immunohistochemistry (IHC) confirmed increased ANXA2 protein expression in melanoma tissues compared with adjacent noncancerous tissues (Figure S3D).

### Preparation and characterization of engineered iEV-150

**Figure 2:**
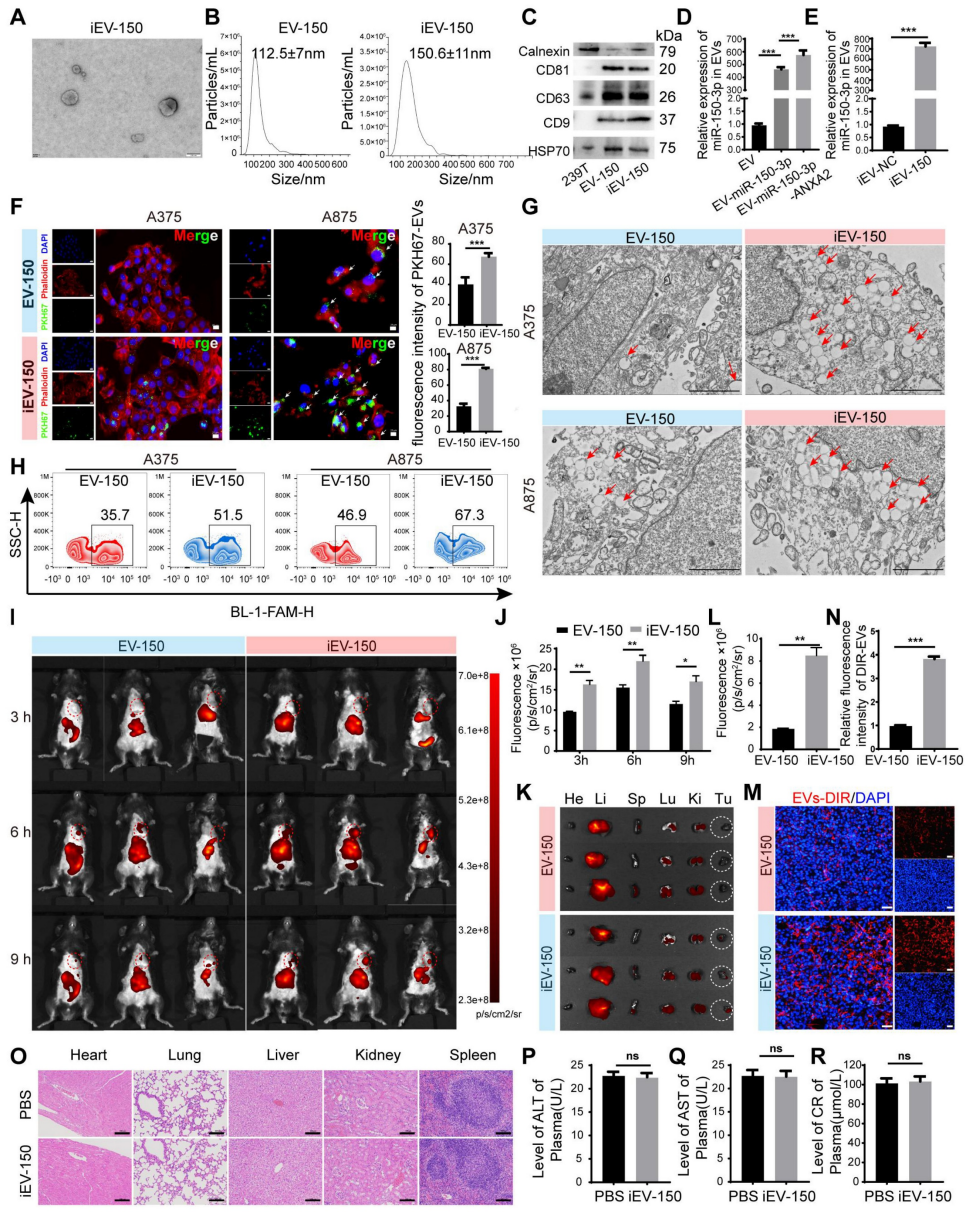
** Preparation of EVs loaded with miR-150-3p and surface modified with iRGD-targeting peptide (iEV-150) and characterization of their enhanced melanoma cell-targeting ability. A** TEM images of engineered EVs (scale: 100 nm). **B** NTA showing the size distribution of engineered EVs. **C** Western blot analysis of EV markers (CD9, CD63, CD81, and HSP70) and a negative marker (calnexin) in EV-150 and iEV-150. **D, E** qRT‒PCR analysis of miR-150-3p levels in EVs from different treatments (D) and in iEV-NC vs. iEV-150 (E). **F** Confocal microscopy of PKH67-labeled EV-150 and iEV-150 uptake by A375 and A875 cells (scale: 25 μm). **G** TEM image showing internalized EV-like structures (red arrows) in melanoma cells after incubation with EV-150 or iEV-150 (scale: 2 μm). **H** Flow cytometry analysis of FAM-labeled EV uptake by melanoma cells. **I** Distribution of DIR-labeled engineered iEV-150 and EV-150 in mouse melanoma models at 3 h, 6 h, and 9 h after tail vein injection (5 × 10^10^ particles), analyzed using an *in vivo* imaging system (IVIS). Each mouse was imaged individually, with 3 mice per group. **J** Analysis of fluorescence intensity in mice *in vivo*. **K** IVIS fluorescence imaging of major organs in mice at 9 h. **L** Fluorescence intensity analysis of mouse tumors. **M, N** Intratumoral distribution of EVs in melanoma tissue sections (scale: 100 μm). **O** H&E staining of major organs after tail vein injection of iEV-150 (5 × 10^10^ particles per dose) or PBS into mice. Injections were administered on Days 7, 10, 13, 16, and 19. Tissues were collected at the endpoint and stained with hematoxylin and eosin (H&E). Scale bar = 100 μm. **P-R** Serum biomarkers of liver (ALT, AST) and kidney (creatinine) function in treated mice (n = 6/group). Mean ± SD, *p < 0.05; **p < 0.01; ***p < 0.001; ns, not significant.

To develop a targeted therapeutic approach for melanoma, we established a stable HEK293T cell line overexpressing miR-150-3p via lentiviral transduction. Additionally, a lentivirus encoding the ANXA2 protein was introduced to further promote the enrichment of miR-150-3p in EVs, as described by Villarroya-Beltri 35 and David Rufino-Ramos 36. Following EV isolation by ultracentrifugation, we conjugated the iRGD peptide to the EV membrane, generating iRGD-EVs-miR-150-3p (iEV-150), which was designed to selectively target melanoma cells. We characterized iEV-150 and control iRGD-EVs-miR-NC (iEV-NC) using transmission electron microscopy (TEM), nanoparticle tracking analysis (NTA), and Western blot analysis. TEM revealed a classic “cup-shaped” morphology for iEV-150 (Figure 2A), whereas NTA revealed mean diameters of 112.5 ± 7 nm for EV-150 and 150.6 ± 11 nm for iEV-150 (Figure 2B). Western blot analysis confirmed the presence of the EV markers CD9, CD63, CD81, and HSP70, with no detectable calnexin expression, in both EV-150 and iEV-150 (Figure 2C).

Quantitative RT‒PCR (qRT‒PCR) analysis revealed a 490-fold increase in miR-150-3p levels in EVs from miR-150-3p-overexpressing cells compared with parental 293T-derived EVs, with further elevation following ANXA2 overexpression (Figure 2D). In iEV-150, miR-150-3p expression was significantly greater than that in iEV-NC, confirming efficient loading (Figure 2E). To further evaluate whether miR-150-3p was indeed encapsulated within the EVs, iEV-150 was treated with PBS (control), RNase A, Triton X-100 (to disrupt the EV membrane), or a combination of RNase A and Triton X-100 (Figure S4A). As expected, treatment with either RNase A or Triton X-100 alone did not affect the detection of miR-150-3p. However, simultaneous treatment with RNase A and Triton X-100 resulted in a 95% reduction in the miR-150-3p level. These findings confirmed that miR-150-3p was encapsulated within iEV-150.

### iEV-150 exhibited enhanced targeting ability toward melanoma cells

To validate the rationale for iRGD-mediated targeting, we performed flow cytometry to assess the surface expression of integrins αvβ3 and αvβ5 by B16-F10 melanoma cells. Overlay histograms revealed markedly greater fluorescence intensities for αvβ3 (blue) and αvβ5 (orange) than for the isotype control (cyan) and unstained cells (red), confirming high expression of both integrins (Figure S4B). To assess the targeting efficacy of engineered EVs toward melanoma cells, we employed laser scanning confocal microscopy (LSCM) to evaluate the uptake of PKH67-labeled EVs by melanoma cell lines (A375, A875, and B16-F10). These cells exhibited significantly greater uptake of iEV-150 than nontargeted EV-150 (Figure 2F and Figure S4C), highlighting the enhanced cellular internalization conferred by iRGD modification. TEM further confirmed that iEV-150 was internalized by melanoma cells in greater quantities than unmodified EVs were (Figure 2G).

To validate the presence and functionality of the iRGD-targeting peptide, we conjugated FAM-labeled Chol-PEG-CRGDKGPDC to EVs and assessed its delivery efficiency to A375, A875, and B16-F10 cells using flow cytometry. FAM fluorescence signals in cells incubated with iEV-150 were markedly elevated compared with those in cells incubated with EV-150, demonstrating that the iRGD peptide significantly enhances iEV-targeting specificity for melanoma cells (Figure 2H and Figure S4D-E).

To investigate the *in vivo* targeting capacity of these engineered EVs, we administered DIR-labeled EV-150 and iEV-150 (5 × 10^10^ particles) via tail vein injection into C57BL/6 mice bearing subcutaneous B16-F10 melanoma tumors. *In vivo* imaging system (IVIS) analysis revealed that, compared with unmodified EV-150, iEV-150 displayed superior tumor-targeting ability (Figure 2I-J). Specifically, the fluorescence intensity of iEV-150 at tumor sites was significantly stronger than that of EV-150, peaking at 6 h postinjection and indicating improved delivery efficiency due to the iRGD peptide. To further quantify its biodistribution, major organs (kidney, liver, lungs, spleen, and heart) and tumors were excised at 9 h postadministration for *ex vivo* imaging. The results demonstrated a pronounced accumulation of iEV-150 at the tumor site compared with that of EV-150 (Figure 2K-L). Examination of tumor sections revealed deeper infiltration of iEV-150 within the tumor parenchyma, with notably stronger fluorescence signals than those observed with EV-150 (Figure 2M-N). Importantly, the qRT‒PCR results revealed that the level of miR-150-3p in melanoma tissues from mice treated with iEV-150 was significantly greater than that in the EV-150 group. This finding indicates that following intravenous injection, iEV-150 successfully delivers miR-150-3p to the tumor tissue rather than losing its cargo in the systemic circulation (Figure S4F).

Safety is a pivotal consideration for the translational potential of EV-based delivery systems. To evaluate systemic toxicity, we administered iEV-150 (5 × 10^10^ particles) intravenously to healthy C57BL/6 mice via tail vein injection every other day for one week. Compared with those in the PBS-treated controls, no mortality or significant body weight loss was observed in the iEV-150 group throughout the study (data not shown). Given that nanoscale lipid vesicles are predominantly cleared by the mononuclear phagocyte system (MPS) following intravenous administration 37, we conducted histopathological analysis of major organs (heart, liver, spleen, lungs, and kidneys) to detect potential adverse effects. Hematoxylin and eosin (H&E) staining revealed no notable pathological abnormalities or inflammatory lesions in the iEV-150-treated group (Figure 2O), suggesting negligible inflammatory responses.

To further assess toxicity, we performed blood biochemistry and hematological analyses, focusing on the liver function markers alanine aminotransferase (ALT; Figure 2P) and aspartate aminotransferase (AST; Figure 2Q), as well as the renal function marker creatinine (CRE; Figure 2R). The levels of these biomarkers in iEV-150-treated mice were comparable to those in PBS-treated controls, indicating that there was no detectable hepatic or renal toxicity at the administered dose. Collectively, these data demonstrate that repeated dosing of iEV-150 elicits no acute toxicity across major organ systems in mice, supporting its safety profile for potential therapeutic applications.

### iEV-150 inhibits melanoma cell proliferation, DNA damage repair, and EMT *in vitro* and suppresses tumor growth and metastasis *in vivo* accompanied by increased melanoma cell death

To explore the therapeutic potential of engineered EVs in melanoma, we cocultured iEV-150 and iEV-NC with melanoma cells (A375 and A875) to investigate their effects on cellular behavior. Initially, we optimized the EV concentration to ensure minimal baseline impact on the melanoma cell phenotype while accurately capturing iEV-150-specific effects. Higher concentrations of iEV-150 (5 × 10^10^ particles/mL) markedly suppressed melanoma cell proliferation (Figure S5A), whereas a baseline concentration of 1 × 10^10^ particles/mL had no significant effect (Figure S5B). Consequently, we selected 5 × 10^10^ particles/mL for subsequent experiments. Coculture with iEV-150 significantly elevated miR-150-3p levels in melanoma cells (Figure S5C), whereas neither iEV-NC nor iEV-150 altered pri-miR-150 expression in A375 or A875 cells (Figure S5D-E). In contrast, transfection with a miR-150-3p inhibitor substantially reduced pri-miR-150 levels in both cell lines (Figure S5D-E). These data indicate that iEV-150 augments miR-150-3p in melanoma cells via EV-mediated delivery rather than by stimulating endogenous expression.

**Figure 3:**
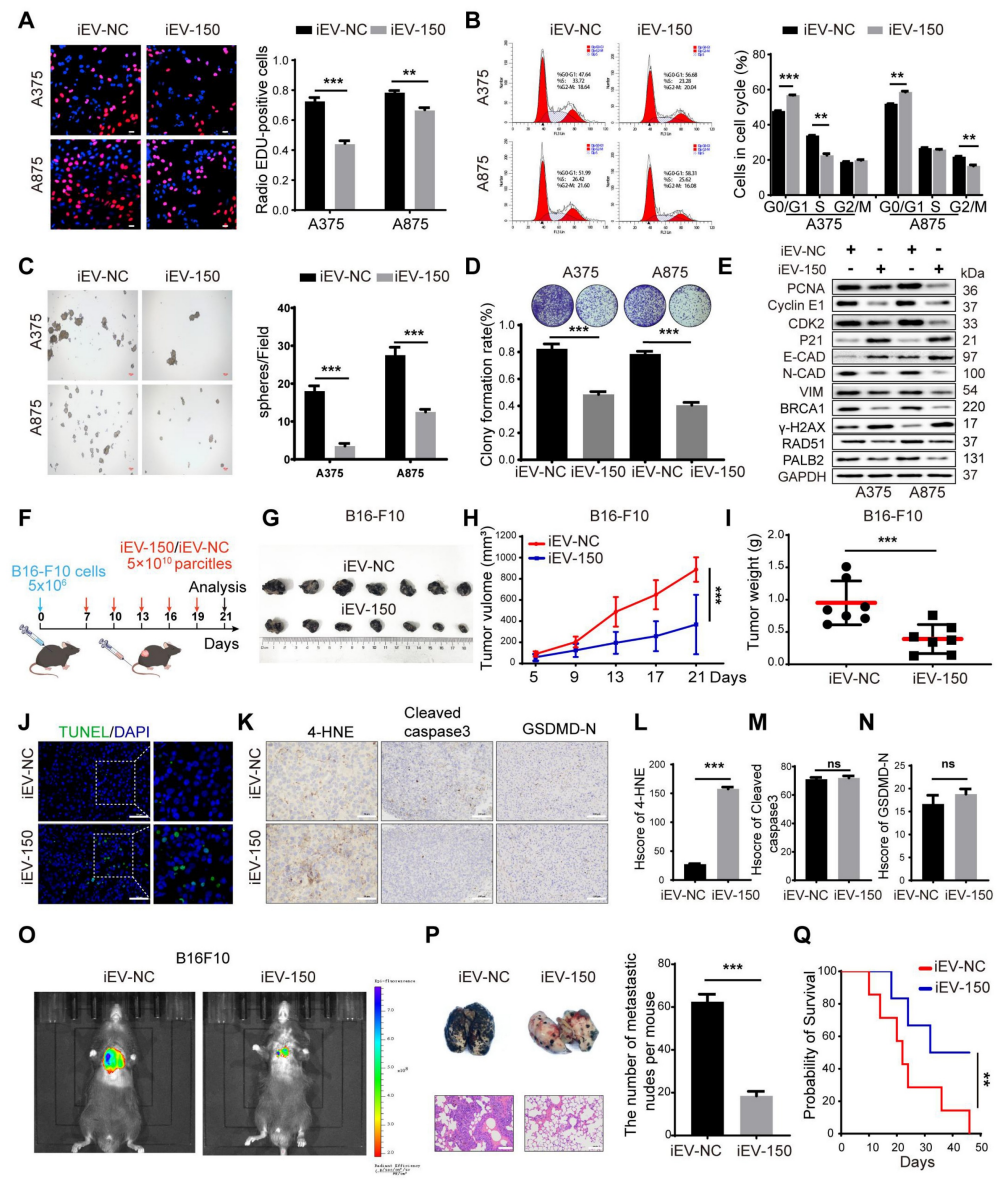
** iEV-150 suppresses melanoma cell proliferation and metastasis while promoting cell death *in vitro* and *in vivo*. A** DNA replication of A375 and A875 cells was determined by EDU staining. Scale bar: 20 μm. **B** Cell cycle of A375 and A875 cells was analyzed by Flowcytometry. **C, D** Sphere formation (scale: 100 μm) (C) and colony formation (D) assays showing growth inhibition by iEV-150. **E** Western blot of proliferation, EMT, and DNA damage repair markers. **F** Schematic diagram of B16-F10 cell subcutaneous injection and engineered EVs tail vein injection in C57/BL6 mice. **G-I** Tumor images (G), growth curves (H), and tumor weights (I) in mice treated with iEV-150 or iEV-NC. **J** TUNEL staining of xenografts showing apoptotic cells (green, scale: 50 μm); quantified from three fields per mouse. **K-N** IHC staining (K, scale: 50 μm) and quantification (L-N) of 4-HNE, cleaved caspase-3, and GSDMD-N in tumor sections. **O** Bioluminescence imaging of B16-F10-luc metastasis after treatment. **P** Lung metastasis model: gross images (top), nodule counts (middle), and H&E staining (bottom, scale: 100 μm). **Q** Kaplan-Meier survival curves of tumor-bearing mice treated with iEV-150 or iEV-NC. Mean ± SD, *p < 0.05; **p < 0.01; ***p < 0.001; ns, not significant.

We then investigated the effects of iEV-150 on DNA replication and cell cycle progression. EdU staining revealed that coculturing melanoma cells with iEV-150 reduced the proportion of EdU-positive cells (Figure 3A). Cell cycle analysis also revealed that iEV-150 coculture decreased the proportion of cells in the S and G2/M phases while increasing the proportion in the G1 phase (Figure 3B). Moreover, sphere formation (Figure 3C) and colony formation (Figure 3D) assays revealed that iEV-150 coculture inhibited melanoma cell proliferation. Mechanistically, iEV-150 downregulated CDK2 and cyclin E2 while upregulating the CDK inhibitor P21, which is involved in the S phase (Figure 3E). iEV-150 also decreased the expression of PCNA, a factor associated with DNA replication (Figure 3E). After iEV-150 coculture, E-cadherin expression increased, whereas N-cadherin and vimentin expression decreased, suggesting the inhibition of epithelial‒mesenchymal transition (EMT) in melanoma cells (Figure 3E). Furthermore, we observed downregulation of the expression of BRCA1, RAD51, and PALB2, key DNA repair factors, and an increase in the expression of the DNA damage marker γ-H2AX (Figure 3E). These findings indicate that iEV-150 reduces the DNA repair capacity of melanoma cells.

To further investigate the effect of iEV-150, we intravenously injected iEV-150 or iEV-NC into mice with established subcutaneous tumors starting on Day 7, with injections given every 3 d (Figure 3F). Compared with the iEV-NC-treated group, the iEV-150-treated group presented significantly reduced tumor growth and weight (Figure 3G-I). Additionally, TUNEL staining revealed an increase in the number of dead cells in the tumor sections from iEV-150-treated mice (Figure 3J), suggesting that iEV-150 may induce melanoma cell death. IHC analysis also revealed a significant increase in the expression of the lipid peroxidation marker 4-hydroxynonenal (4-HNE) in the tumors of iEV-150-treated mice, indicating increased lipid peroxidation (Figure 3K-L). There were no significant differences in the staining of apoptosis markers, cleaved caspase-3, or the N-terminal domain of pyroptotic executor GSDMD (GSDMD-N) between tumors treated with iEV-150 and iEV-NC (Figure 3K and Figure 3M-N). In addition, iEV-150 reduced the number of lung nodules induced by B16-F10 in mice (Figure 3O-P). Survival analysis revealed that the mice in the iEV-150 group lived longer than those in the iEV-NC group did (Figure 3Q). Collectively, these results suggest that iEV-150 plays an essential role in regulating melanoma proliferation, DNA damage repair, EMT, and mouse survival.

### iEV-150 inhibits the proliferation and metastasis of melanoma cells by promoting ferroptosis

**Figure 4:**
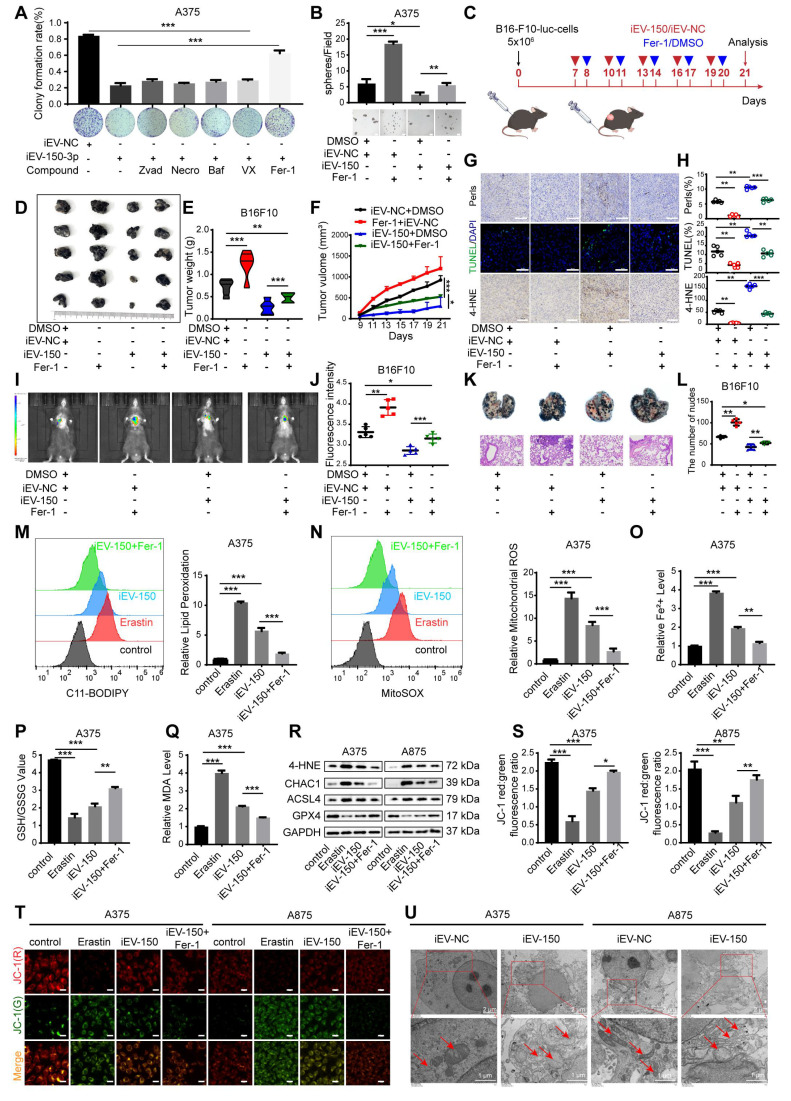
** iEV-150 induces ferroptosis and inhibits melanoma growth and metastasis. A** A375 cells co-cultured with iEV-150 were treated with RCD inhibitors—z-VAD-FMK (20 μM), Nec-1 (10 μM), Baf-A1 (70 nM), VX-765 (10 μM), or Fer-1 (5 μM)—and cell proliferation was assessed after 3 d. **B** Spheroid formation of A375 cells in different treatment groups. **C** Schematic of *in vivo* experiment: B16-F10 cells were subcutaneously injected, followed by tail vein injection of iEV-150 or iEV-NC combined with Fer-1 (5 mg/kg, prepared as a 5 mmol/L stock in DMSO) or vehicle. D-F: Tumor images (D), weights (E), and growth curves (F) in each group. **G** Prussian blue, TUNEL, and 4-HNE IHC staining of tumor sections (scale: 25 μm). **H** Quantification of staining results. **I, J** IVIS imaging and quantification of lung fluorescence signals. **K, L** Representative lung metastasis images and H&E staining (K), with nodule quantification (L). **M, N** Lipid ROS and mitochondrial superoxide levels in A375 cells from indicated treatment groups. **O**-**Q** Quantification of Fe²⁺, GSH/GSSG ratio, and MDA levels. **R** Western blot of ferroptosis-related proteins (4-HNE, CHAC1, ACSL4, GPX4). **S, T** JC-1-based analysis and images of mitochondrial membrane potential in A375 and A875 cells (scale: 10 μm). **U** TEM showing mitochondrial ultrastructure after iEV-NC or iEV-150 treatment (scale: 2 μm). Mean ± SD, *p < 0.05; **p < 0.01; ***p < 0.001.

Several forms of regulated cell death (RCD), including apoptosis, necrosis, pyroptosis, ferroptosis, and autophagy-dependent cell death, are well characterized. We next sought to determine whether iEV-150 inhibits melanoma growth by inducing any of these forms of RCD. To verify this, we employed five distinct RCD-specific small-molecule inhibitors to selectively block individual cell death pathways. After treatment with ferrostatin-1 (Fer-1), an inhibitor of ferroptosis, cell death in the iEV-150 coculture group was significantly reduced, suggesting that iEV-150 may partially inhibit cell growth by inducing ferroptosis (Figure 4A-B and Figure S6A-C). Tail vein injection of iEV-150 significantly reduced tumor size, growth, and weight in mice with subcutaneous melanoma. However, compared with iEV-150 treatment alone, coadministration of iEV-150 and ferrostatin-1 partially restored tumor size and weight (Figure 4C-F). Prussian blue staining of tumor sections revealed elevated iron levels in the iEV-150 group, whereas coadministration of Fer-1 reduced iron levels (Figure 4G-H). Similarly, TUNEL staining revealed increased cell death in the iEV-150 group, which was attenuated by ferrostatin-1 treatment (Figure 4G-H). A similar trend was observed for 4-HNE levels (Figure 4G-H). Additionally, we found that the reduction in the lung metastasis of melanoma cells in mice caused by iEV-150 treatment was partially reversed by ferrostatin-1 (Figure 4I-L).

To confirm these findings, we used the ferroptosis inducer erastin and the ferroptosis inhibitor Fer-1 to assess intracellular lipid ROS levels (Figure 4M and Figure S6D), mitochondrial superoxide levels (Figure 4N and Figure S6E), Fe²⁺ levels (Figure 4O and Figure S6F), the GSH/GSSG ratio (Figure 4P and Figure S6G) and MDA levels (Figure 4Q). As expected, erastin and iEV-150 treatment increased intracellular lipid ROS, mitochondrial superoxide, Fe²⁺, MDA, and cell death levels while reducing the GSH/GSSG ratio. These effects were reversed by Fer-1. Additionally, both erastin and iEV-150 upregulated 4-HNE, CHAC1, and ACSL4 while downregulating GPX4, and Fer-1 partially reversed these changes (Figure 4R).

The red-to-green fluorescence ratio in JC-1-stained cells reflects the mitochondrial membrane potential (MMP). In healthy mitochondria, JC-1 forms red fluorescent aggregates, while depolarized mitochondria exhibit green fluorescence. After 24 h of erastin or iEV-150 treatment, the red/green fluorescence ratio decreased, indicating a decrease in the MMP. This decrease was partially reversed by Fer-1 (Figure 4S-T). Next, we used transmission electron microscopy (TEM) to examine the cellular ultrastructure. As shown in Figure 4U, compared with those in iEV-NC-treated cells, the mitochondria in A375 and A875 cells treated with iEV-150 appeared smaller in size, with increased membrane density and fewer cristae. In summary, our findings indicate that iEV-150 induces ferroptosis and inhibits melanoma growth and metastasis, suggesting its potential as a therapeutic agent for melanoma.

Since iEV-150 also induces cell cycle arrest, we next investigated whether ferroptosis mediates this effect. Ferroptosis inhibition had little effect on cell cycle arrest, suggesting that cell cycle regulation in iEV-150-treated melanoma cells is independent of ferroptosis (Figure S6H). Given that apoptosis is a key mechanism of tumor cell death, we further examined whether it contributes to iEV-150-induced cell death. Our data revealed that iEV-150 had no significant effect on melanoma apoptosis (Figure S6I). Overall, these findings suggest that engineered iEV-150 has good potential as a melanoma therapy.

### iEV-150 inhibits the proliferation and metastasis of melanoma by targeting NF2

**Figure 5:**
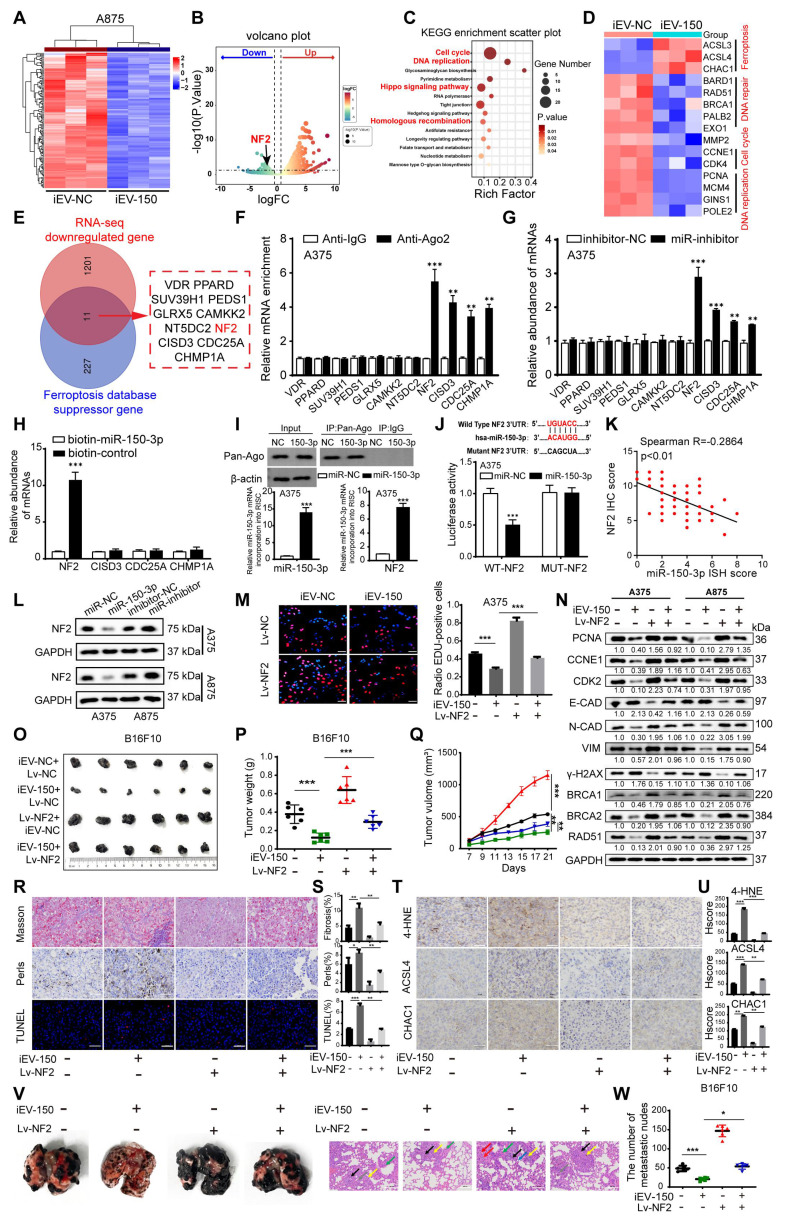
** iEV-150 inhibits the proliferation and metastasis of melanoma by regulating NF2. A, B** Heatmap (A) and volcano plot (B) of downregulated genes in A875 cells after iEV-150 treatment (RNA-seq; p < 0.05, |log₂FC| > 1). **C** KEGG enrichment analysis of differentially expressed genes. **D** Heatmap showing RNA-seq changes in genes related to DNA replication, cell cycle, migration, DNA repair, and ferroptosis. **E** Venn diagram showing overlap between ferroptosis suppressor genes and downregulated RNA-seq genes. **F** AGO2-RIP-qRT-PCR analyzed mRNA enriched with miR-150-3p. **G** qRT-PCR to detect changes in candidate mRNA expression after inhibition of miR-150-3p. **H** miRNA pulldown assay. **I** In miR-150-3p-overexpressing A375 cells, Pan-Ago2 antibody was used for RISC immunoprecipitation. qRT-PCR showed increased enrichment of miR-150-3p and NF2. U6 and GAPDH served as internal controls; IgG and β-actin were used as negative and loading controls, respectively. **J** Luciferase assay validating direct binding of miR-150-3p to NF2 3′UTR. **K** Correlation between miR-150-3p and NF2 expression in melanoma tissue microarrays. **L** Western blot of NF2 expression after miR-150-3p overexpression or inhibition. **M** EDU assay of cell proliferation (scale: 20 μm). **N** Western blot of proliferation, EMT, and DNA repair markers after iEV-150 treatment or NF2 overexpression. O-Q: Tumor images (O), weights (P), and growth curves (Q) in C57BL/6 mice with different treatments. **R, S** Mason trichrome, Prussian blue, and TUNEL staining of tumor sections (R) with quantitative analysis (S); scale: 50 μm. **T, U** IHC of 4-HNE, ACSL4, and CHAC1 in tumor tissues (T) and quantification (U); scale: 50 μm. **V, W** Images (V) and quantification (W) of lung metastases and H&E staining in mice injected with different EV or cell treatments. Mean ± SD, *p < 0.05; **p < 0.01; ***p < 0.001.

To elucidate how iEV-150 promotes ferroptosis and suppresses the malignant phenotype of melanoma cells, we performed RNA sequencing (RNA-seq) on A875 cells cocultured with iEV-150 or iEV-NC. The differentially expressed genes (DEGs) with |log₂FC| > 1 and p < 0.05 (Figure 5A-B) were identified. KEGG pathway enrichment analysis revealed that these DEGs were predominantly associated with cell cycle regulation, DNA replication, DNA damage repair, and the Hippo signaling pathway (Figure 5C). The RNA-seq data further revealed that iEV-150 upregulated the ferroptosis-related genes ACSL3, ACSL4, and CHAC1 while downregulating genes associated with DNA damage repair, cell migration, cell cycle progression, and DNA replication (Figure 5D).

To pinpoint miR-150-3p target genes, we intersected downregulated DEGs from iEV-150-treated A875 cells with ferroptosis inhibitor genes from a ferroptosis database and identified 11 candidates (Figure 5E). Analysis of the TCGA-SKCM and GTEx datasets revealed elevated expression of NF2, SUZ39H1, CDC25A, GLRX5, and CISD3 in melanoma tissues, in contrast with the reduced expression of VDR, PPARD, CAMKK2, NT5DC2, and CHP1A (Figure S7A). AGO2-RIP-qRT‒PCR assays demonstrated that the NF2 and CISD3 mRNAs were markedly enriched in the AGO2-miR-150-3p complex (Figure 5F and Figure S7B), a finding corroborated by their upregulation in miR-150-3p knockout melanoma cells (Figure 5G and Figure S7C). RNA pulldown assays confirmed a robust interaction between miR-150-3p and NF2 (Figure 5H and Figure S7D). RNA-ChIP analysis further validated this finding by showing enhanced NF2 mRNA enrichment in the Ago2/RNA-induced silencing complex (RISC) upon miR-150-3p overexpression (Figure 5I and Figure S7E), with qRT‒PCR confirming elevated miR-150-3p and NF2 levels in RISCs from overexpressing cells (Figure 5I and Figure S7E).

Luciferase reporter assays using plasmids with wild-type or mutant miR-150-3p binding sites in the NF2 3'UTR revealed that miR-150-3p overexpression significantly reduced the luciferase activity of the wild-type construct but not the mutant construct, confirming direct targeting of the NF2 3'UTR (Figure 5J and Figure S7F). IHC analysis revealed elevated NF2 expression in melanoma tissues compared with adjacent normal tissues, with higher levels in stage III/IV patients than in stage I/II patients (Figure S7G-H). Correlation analysis revealed a negative association between miR-150-3p and NF2 expression in melanoma tissues (Figure 5K), whereas survival analysis revealed that high NF2 expression was associated with reduced overall survival (Figure S7I). Notably, patients with high miR-150-3p and low NF2 expression had the best outcomes, whereas those with low miR-150-3p and high NF2 expression had the worst outcomes (Figure S7J).

Further experiments demonstrated that miR-150-3p overexpression suppressed NF2 at both the mRNA and protein levels (Figure 5L and Figure S7K), whereas ANXA2 overexpression increased NF2 expression in melanoma cells (Figure S7L). In NF2-overexpressing melanoma cells cocultured with iEV-150, the results of the EdU and CCK-8 assays revealed that NF2 overexpression counteracted the suppression of proliferation induced by iEV-150 (Figure 5M and Figure S7M). Flow cytometry revealed that NF2 overexpression reversed the iEV-150-mediated shift from the S phase to the G0/G1 phase (Figure S7N), suggesting that iEV-150 inhibits proliferation via NF2 suppression. Western blot analysis of A375 and A875 cells confirmed that NF2 overexpression restored the iEV-150-induced downregulation of the expression of the proliferation marker PCNA, the cell cycle regulators CCNE1 and CDK2, EMT markers (E-cadherin, N-cadherin, and vimentin), the DNA repair genes BRCA1, BRCA2 and RAD51, and the DNA damage marker γ-H2AX (Figure 5N). *In vivo*, NF2 overexpression abrogated the iEV-150-induced reduction in tumor volume and weight in the mice (Figure 5O-Q). Masson staining indicated that the iEV-150-induced increases in muscle fiber content within melanoma tissues were diminished by NF2 overexpression (Figure 5R-S). Prussian blue staining revealed that iEV-150-driven iron accumulation in tumors was reduced by NF2 overexpression (Figure 5R-S), and TUNEL staining confirmed that NF2 overexpression attenuated iEV-150-induced tumor cell death (Figure 5R-S). IHC analysis further revealed that iEV-150 suppressed the expression of the ferroptosis markers 4-HNE, ACSL4, and CHAC1 *in vivo* by inhibiting NF2, and this effect was reversed by NF2 overexpression (Figure 5T-U). Additionally, NF2 overexpression restored iEV-150-suppressed lung metastasis of B16-F10 melanoma in mice (Figure 5V-W). Collectively, these results demonstrate that iEV-150 regulates melanoma malignancy and ferroptosis by directly targeting NF2.

### iEV-150 disrupts NF2-LATS1 binding to inhibit Hippo signaling and promote ferroptosis in melanoma

**Figure 6:**
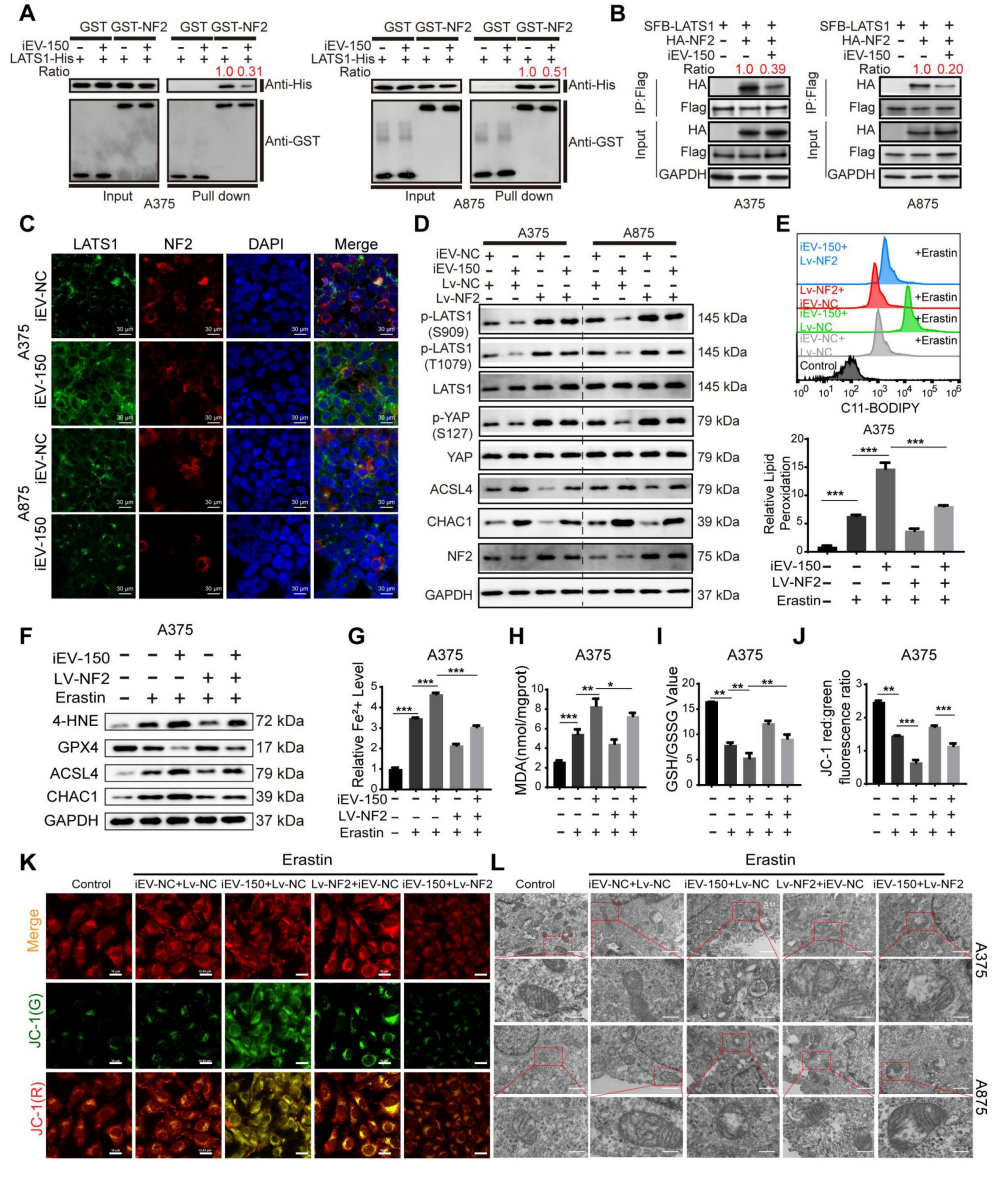
** iEV-150 interacts with NF2, leading to the inactivation of LATS1 kinase and promoting ferroptosis in cells. A, B** GST pulldown (A) and co-IP (B) assays showing decreased NF2-LATS1 binding after iEV-150 treatment.** C** Immunofluorescence detection of NF2-LATS1 interaction in melanoma cells after iEV-150 co-culture (scale: 30 μm). **D** Western blot analysis of ACSL4, CHAC1, and Hippo pathway components in A375 and A875 cells after iEV-150 treatment or NF2 overexpression. **E** Lipid ROS levels analyzed by C11-BODIPY staining and flow cytometry.** F** Western blot of ferroptosis-related proteins in A375 cells treated with Erastin (15 μM), iEV-150, NF2 overexpression, or combinations. **G-L** Quantification of Fe²⁺ (G), MDA (H), GSH/GSSG ratio (I), mitochondrial membrane potential (J, K), and mitochondrial morphology (L) under different treatments. Mean ± SD, *p < 0.05; **p < 0.01; ***p < 0.001.

NF2, an upstream regulator of the Hippo signaling pathway, facilitates the phosphorylation and activation of large tumor suppressor kinase 1 (LATS1), thereby modulating YAP/TAZ activity 38. To explore the impact of iEV-150 on this regulatory axis, we assessed its effect on the NF2-LATS1 interaction. GST pulldown and coimmunoprecipitation (co-IP) assays revealed that iEV-150 significantly diminished the physical interaction between NF2 and LATS1 in melanoma cells (Figure 6A-B). Consistently, immunofluorescence (IF) analysis revealed reduced colocalization of NF2 and LATS1 in iEV-150-treated cells (Figure 6C), confirming the disruption of this interaction.

Next, we investigated the role of the iEV-150-NF2 axis in coordinating ferroptosis and Hippo signaling in melanoma cells. Western blot analysis revealed that coculture with iEV-150 significantly reduced the phosphorylation of LATS1 and YAP in melanoma cells, whereas the total protein levels of LATS1 and YAP remained unchanged (Figure 6D). Concurrently, the expression of the ferroptosis-related markers ACSL4 and CHAC1 was markedly elevated (Figure 6D). These findings suggest that iEV-150 inhibits Hippo pathway activity and promotes ferroptosis-associated gene expression.

To further investigate the role of NF2, we overexpressed NF2 in iEV-150-treated melanoma cells. Notably, NF2 overexpression restored LATS1 and YAP phosphorylation to higher levels without altering their total protein abundance while concomitantly reducing the expression of ACSL4 and CHAC1 (Figure 6D). These results indicate that iEV-150 suppresses Hippo signaling and enhances ferroptosis by targeting NF2, an effect that is reversed by NF2 overexpression, which reactivates the pathway and attenuates ferroptosis induction.

To further delineate the ferroptosis-modulating effects of this axis, we evaluated melanoma cells treated with the ferroptosis inducer erastin alongside iEV-150, with or without NF2 overexpression. Comprehensive profiling of ferroptosis hallmarks, including lipid peroxidation levels (Figure 6E and Figure S8A), ferroptosis-regulating proteins (Figure 6F and Figure S8B), Fe²⁺ accumulation (Figure 6G and Figure S8C), MDA levels (Figure 6H and Figure S8D), the GSH/GSSG ratio (Figure 6I and Figure S8E), JC-1 fluorescence intensity reflecting the mitochondrial membrane potential (Figure 6J-K and Figure S8F), and the mitochondrial ultrastructure assessed by TEM (Figure 6L), demonstrated that iEV-150 significantly enhanced ferroptosis signatures, comparable to the effects of erastin. Notably, NF2 overexpression in iEV-150-treated cells partially mitigated these effects, reducing lipid peroxidation, Fe²⁺ levels, and mitochondrial damage while restoring the GSH/GSSG balance and the MMP. These findings collectively demonstrate that iEV-150 promotes ferroptosis in melanoma cells by suppressing NF2 expression, which disrupts NF2-LATS1 binding, inhibits Hippo signaling, and promotes ferroptosis induction.

### iEV-150 induces ferroptosis through YAP modulation in the Hippo signaling pathway by regulating CHAC1 and ACSL4

We then explored the interplay between ferroptosis mediated by the iEV-150-NF2 axis and the Hippo-YAP pathway. As previously demonstrated, iEV-150 coculture reduced the phosphorylation of LATS1 (Ser909, Thr1079) and YAP (Ser127) while increasing CHAC1 and ACSL4 expression (Figure 6D). Immunofluorescence (Figure S9A) and Western blot (Figure S9B) analyses revealed that iEV-150 induced YAP translocation from the cytoplasm to the nucleus in melanoma cells. Strikingly, in YAP-knockout A375 and A875 cells, iEV-150 coculture failed to increase ACSL4 and CHAC1 expression (Figure S9C-D). The re-expression of YAP restored these markers to levels comparable to those in iEV-150-treated controls (Figure S9E). Consistently, assessments of lipid peroxidation (Figure S9F-G), the cell death rate (Figure S9H), MDA levels (Figure S9I), Fe²⁺ accumulation (Figure S9J), the GSH/GSSG ratio (Figure S9K), mitochondrial morphology via TEM (Figure S9L), and JC-1 fluorescence intensity (Figure S9M) revealed that YAP knockout abolished iEV-150-induced ferroptosis, an effect reversed by YAP re-expression (Figure S10A-G). These data underscore the pivotal role of the Hippo-YAP pathway in iEV-150-mediated ferroptosis regulation.

Notably, the iEV-150-mediated upregulation of ACSL4 and CHAC1 is governed by YAP-dependent regulation. ACSL4 is a known downstream target of YAP, so we investigated whether YAP directly controls CHAC1 transcription. Luciferase assays with CHAC1 promoter mutants spanning 250 nt upstream of the transcription start site (TSS) revealed that deleting the -500 to -250-nt region reduced basal promoter activity to levels similar to those of the pGL3 control (Figure S11A), indicating a critical YAP-binding region. Bioinformatics analysis predicted a YAP/TEAD4 putative binding site (PBS) within this -500 to -250 nt segment (Figure S11B). A CHAC1 promoter reporter with a precise PBS deletion (Del-YAP) exhibited complete loss of activity (Figure S11C). Transfection with sg-YAP suppressed CHAC1 promoter activity, whereas Flag-YAP overexpression significantly enhanced CHAC1 promoter activity (Figure S11D); notably, these effects were absent in the Del-YAP construct (Figure S11D). ChIP assays confirmed the direct binding of YAP to the CHAC1 promoter at the PBS, with no occupancy detected 2 kb upstream of the TSS (Figure S11E). These findings establish that YAP directly regulates CHAC1 transcription by binding to its promoter.

### The coculture of iEV-150 with melanoma cells promoted the function of CD8+ T cells

**Figure 7:**
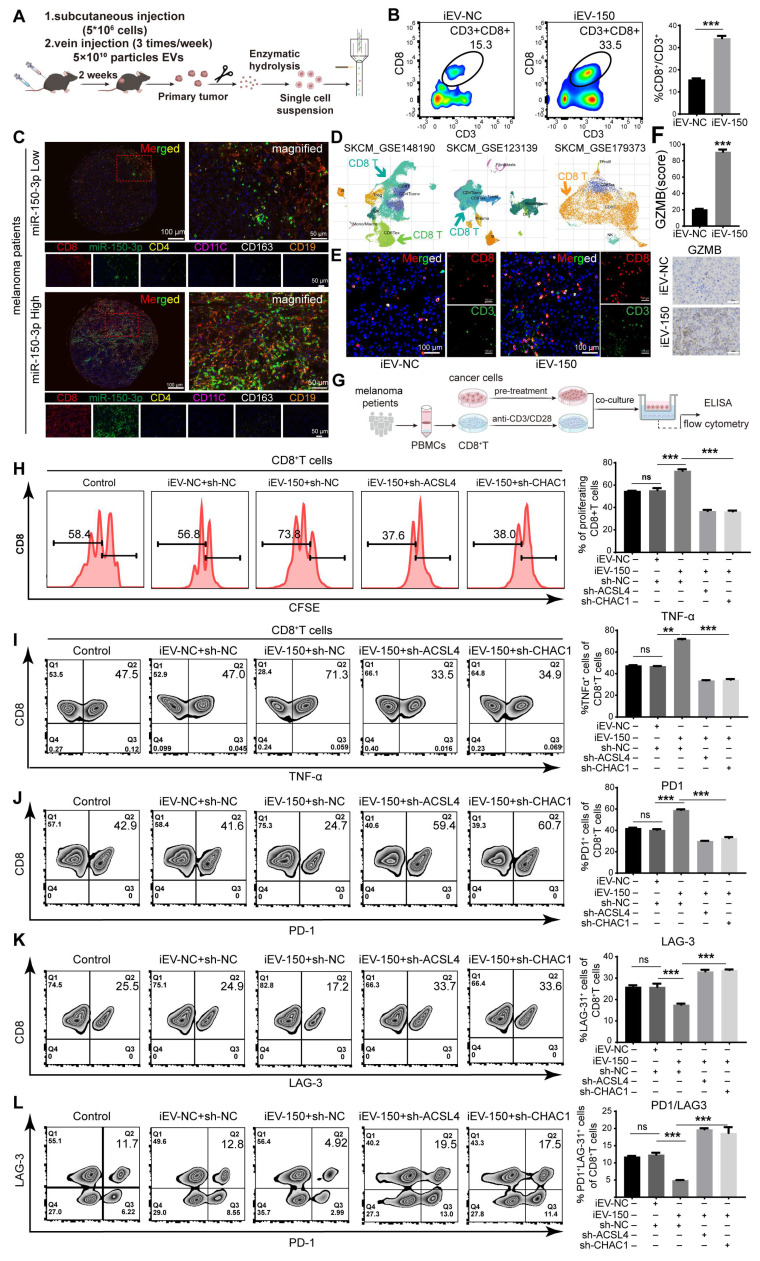
** iEV-150-treated melanoma cells enhance CD8⁺ T cell function. A** Schematic of tumor dissociation and flow cytometry in iEV-NC- or iEV-150-treated mice. **B** Flow cytometry analysis of CD8+ T cell infiltration in mouse tumor tissues. **C** Multiplex immunofluorescence of immune cell populations in melanoma tissues with low vs. high miR-150-3p expression. **D** Immune cell infiltration analysis from single-cell datasets (GSE148190, GSE123139, GSE179373). **E** IF staining of CD3 and CD8 in mouse tumors (scale: 100 μm). **F** IHC staining of granzyme B in mouse tumor tissues. **G** Schematic of PBMC isolation, CD8⁺ T cell purification, and co-culture with A375 cells pretreated with iEV-150 or sh-ACSL4/sh-CHAC1. **H** CFSE-based flow cytometry of CD8⁺ T cell proliferation. **I**-**L** Flow cytometry analysis of TNF-α (I), PD-1 (J), LAG-3 (K), and PD-1⁺LAG-3⁺ (L) expression in CD8⁺ T cells after co-culture. Mean ± SD, *p < 0.05; **p < 0.01; ***p < 0.001.

Bioinformatics analysis revealed a significant correlation between miR-150-3p expression and various immune cell populations in melanoma patients (Figure S12A-C). Therefore, we investigated the effects of iEV-150 treatment on immune cell infiltration within mouse tumor tissues using flow cytometry (Figure 7A). Our results demonstrated that iEV-150 treatment markedly increased the proportion of tumor-infiltrating CD8+ T cells (Figure 7B) but had no notable effect on other immune cell subsets (Figure S12D-G). Multiplex immunofluorescence (mIF) analysis further confirmed that melanoma patients with elevated miR-150-3p expression exhibited significantly greater CD8+ T-cell infiltration in tumor tissues than did those with lower expression levels (Figure 7C). Analysis of three single-cell RNA sequencing datasets from melanoma samples (GSE148190, GSE123139, and GSE179373) consistently supported this enhanced CD8+ T-cell infiltration pattern (Figure 7D and Figure S13A). Immunofluorescence (IF) and IHC analyses further revealed increased CD8+ T-cell infiltration and elevated Granzyme B expression in tumors from iEV-150-treated mice (Figure 7E-F). Collectively, these data indicate that iEV-150 treatment may remodel the tumor microenvironment, promoting CD8+ T-cell recruitment and enhancing the efficacy of T-cell-mediated immunotherapy.

Given the critical role of CD8+ T cells as effector cells in the melanoma microenvironment and their association with prognosis, we next examined whether iEV-150 modulates their functionality. CD8+ T cells were isolated from the peripheral blood mononuclear cells (PBMCs) of 16 melanoma patients and activated with CD3/CD28/IL-2 stimulation, with CD45+/CD3+/CD8+ cells identified as the CD8+ T-cell population (Figure S13B). A375 melanoma cells were subsequently pretreated with iEV-NC, iEV-150, or iEV-150 combined with sh-ACSL4 or sh-CHAC1 for 48 h before being cocultured with CD8+ T cells (Figure 7G). The results of the coculture experiments revealed that CD8+ T-cell proliferation was significantly enhanced in the presence of iEV-150-treated A375 cells, whereas this effect was attenuated by sh-ACSL4 or sh-CHAC1 cotreatment (Figure 7H). Additionally, flow cytometry and ELISA revealed elevated TNF-α, IFN-γ, granzyme B (GZMB), and perforin levels in CD8+ T cells from the iEV-150 treatment group (Figure 7I and Figure S14A-D) and decreased PD-1 and LAG-3 expression (Figure 7J-K and Figure S14A). This shift, marked by a reduced PD-1-to-LAG-3 expression ratio and fewer PD-1⁺LAG-3⁺ cells (Figure 7L), indicates diminished T-cell exhaustion and a robust antitumor phenotype. Notably, sh-ACSL4 or sh-CHAC1 treatment reversed these effects (Figure 7I-L and Figure S14A-D), confirming the ferroptosis-mediated impact of iEV-150 on CD8+ T-cell activity.

### Increased expression of miR-150 is associated with better clinical outcomes in melanoma patients and is linked to the response to immunotherapy

**Figure 8:**
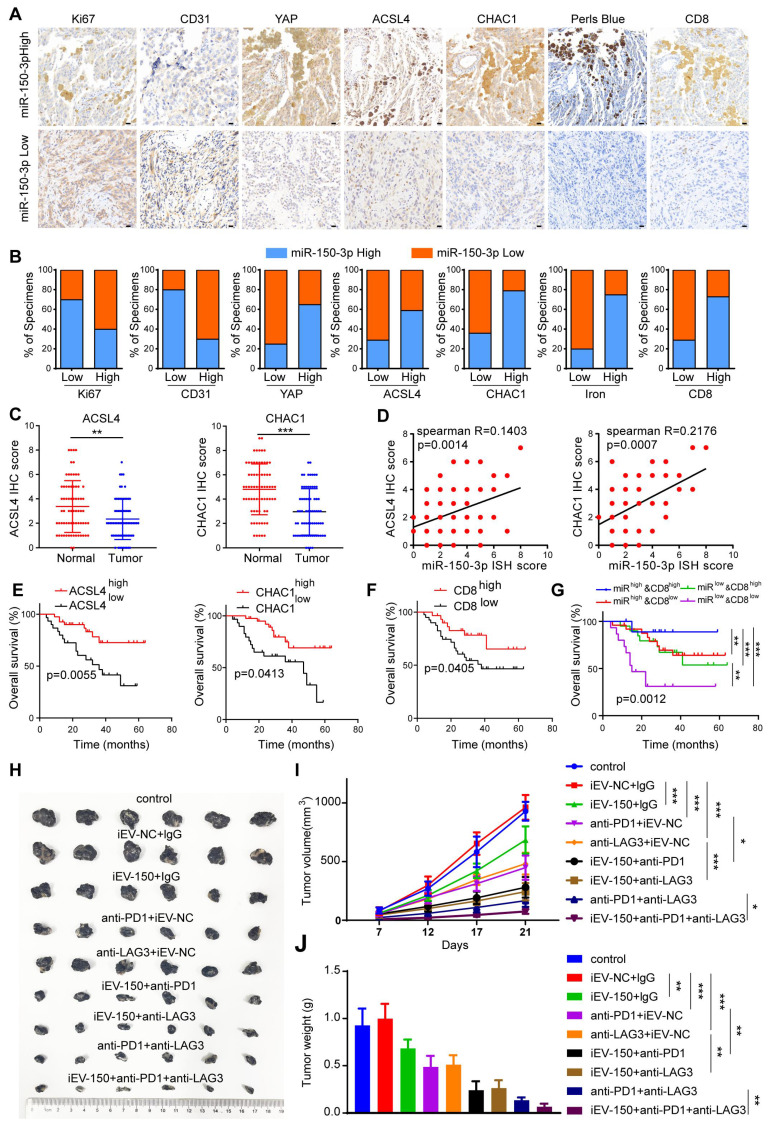
** Increased expression of miR-150 is associated with better clinical outcomes in melanoma patients and is linked to the response to immunotherapy. A** IHC staining of Ki67, CD31, YAP, ACSL4, CHAC1, CD8, and Prussian blue staining in tissues with low vs. high miR-150-3p expression (scale: 20 μm). **B** Quantification of marker expression and iron levels in high vs. low miR-150-3p groups (χ² test). **C** IHC analysis of ACSL4 and CHAC1 expression in melanoma tissue microarrays. **D** Correlation analysis of miR-150-3p with ACSL4 and CHAC1 expression in melanoma patient tissues. **E-G** Survival analysis based on ACSL4/CHAC1 (E), CD8⁺ T cell infiltration (F), and combined miR-150-3p and CD8⁺ expression (G). **H-J** Tumor images (H), growth curves (I), and tumor weights (J) in mice treated with iEV-150 plus anti-PD-1 and anti-LAG3. Mean ± SD, *p < 0.05; **p < 0.01; ***p < 0.001.

Given the close functional relationship between iEV-150 and YAP in promoting ferroptosis and inhibiting tumor growth, they may be involved in melanoma progression from a pathological perspective. To verify this hypothesis, we analyzed miR-150-3p expression in 70 melanoma tissue samples using ISH. The samples were categorized into miR-150-3p low or miR-150-3p high groups on the basis of the median expression level. We then performed IHC to assess the clinical relevance of miR-150-3p with respect to tumor proliferation, angiogenesis, ferroptosis, and CD8+ T-cell infiltration in melanoma tissues (Figure 8A). Our results revealed that miR-150-3p expression was negatively correlated with the expression of Ki67 (a proliferation marker) and CD31 (an angiogenesis marker) in melanoma samples (Figure 8A-B). In contrast, miR-150-3p expression was positively correlated with YAP, ACSL4, CHAC1, and CD8+ T-cell infiltration and iron accumulation in melanoma tissues (Figure 8A-B). Furthermore, ACSL4 and CHAC1 expression levels were significantly lower in melanoma tissues than in matched normal samples (Figure 8C). Both ACSL4 and CHAC1 were positively correlated with miR-150-3p expression (Figure 8D), and their low expression levels were associated with poor survival outcomes in melanoma patients in an independent cohort (Figure 8E). Interestingly, increased CD8+ T-cell infiltration was associated with better outcomes of melanoma patients (Figure 8F). Notably, patients in the CD8-high/miR-150-3p-high group had the best survival outcomes, whereas those in the CD8-low/miR-150-3p-low group had the worst outcomes (Figure 8G).

Next, we explored the impact of iEV-150 on the efficacy of anti-PD-1 and anti-LAG3 immunotherapies. Compared with the control and iEV-NC groups, melanoma-bearing mice treated with iEV-150 showed enhanced antitumor responses when receiving the same dose of anti-PD-1 or anti-LAG3 antibodies, as reflected by reduced tumor size and weight (Figure 8H-J). Notably, combining anti-PD-1 therapy with anti-LAG3 therapy was more effective than either treatment alone. Most notably, the triple combination of iEV-150, anti-PD-1, and anti-LAG3 had the strongest antitumor effects on melanoma-bearing mice (Figure 8H-J). In summary, these findings suggest that iEV-150 promotes ferroptosis in melanoma, inhibits tumor proliferation, and enhances tumor cell sensitivity to anti-PD-1 and anti-LAG3 immunotherapy.

## Discussion

In this study, we developed engineered EVs (iEV-150) by modifying their membranes with iRGD peptides to enhance tumor targeting and therapeutic efficacy. Our findings demonstrate that iEV-150 efficiently delivers miR-150-3p to melanoma cells, induces ferroptosis via the NF2-Hippo-YAP axis, and reprograms the tumor immune microenvironment (TME) by enhancing CD8+ T-cell infiltration and activation. These findings indicate that iEV-150 is a promising dual-function biotherapeutic for RNA-based melanoma treatment that combines direct tumor inhibition with immune modulation.

Unlike previous studies that passively explored tumor-derived EVs as natural miRNA carriers, our approach rationally engineers EVs to optimize miRNA loading, tumor selectivity, and functional stability. One major distinction between our study and prior EV-based melanoma therapies lies in the mechanistic enhancement of miRNA packaging. Previous studies have demonstrated that RBPs such as MVP 39, hnRNPA2B1 40, and SRSF1 41 play roles in the sorting of miRNAs into EVs.

However, our study identified ANXA2 as a novel regulator of miR-150-3p packaging, providing direct evidence that ANXA2 selectively enhances miR-150-3p encapsulation in melanoma-derived EVs. The overexpression of ANXA2 significantly increased the level of miR-150-3p in EVs while reducing its intracellular content, whereas ANXA2 knockout blocked miR-150-3p export and suppressed melanoma proliferation, suggesting that melanoma cells actively secrete miR-150-3p via EVs as a tumor-evasion strategy. This contrasts with glioblastoma studies, where hnRNPA2B1 dominated miRNA sorting, underscoring cancer type-specific mechanisms in EV-mediated miRNA transport 42. Although ANXA2 may have broader RNA-binding capacity, our findings highlight its specific and biologically relevant interaction with miR-150-3p in melanoma cells. This selectivity is supported by our RIP and pulldown assays, which consistently demonstrated predominant enrichment of miR-150-3p under our experimental conditions.

Our study further distinguished itself through its tumor-targeting strategy. Previous studies, such as Tong *et al.* 42 (IMTP-conjugated EVs for cardiac delivery) and Zhou *et al.* 15 (integrin α5-targeted EVs for pancreatic CAF targeting), improved EV biodistribution, they did not address miRNA cargo optimization. In contrast, we combined ANXA2-enhanced miR-150-3p loading with iRGD peptide functionalization, achieving both precise melanoma targeting and potent therapeutic delivery. The iRGD modification facilitates a sequential binding mechanism—initial integrin binding followed by proteolytic unmasking of a CendR motif and interaction with NRP-1—that enables active internalization beyond membrane-level anchoring 43, 44.

Unlike previous iRGD-modified melanoma therapies, which primarily utilized synthetic liposomes or polymeric nanoparticles to target αvβ3 integrins in the tumor vasculature 45-47, our EV-based approach offers superior biocompatibility and stability. Synthetic lipid nanoparticles (LNPs), for example, often struggle with immune clearance and systemic stability, limitations that iEV-150 overcomes by leveraging the natural properties of EVs.

Our study reveals a novel mechanism by which iEV-150 induces ferroptosis in melanoma cells through the NF2-Hippo-YAP signaling axis. Specifically, iEV-150 downregulates NF2, disrupting its interaction with LATS1 and thereby inhibiting Hippo pathway activity. This leads to YAP nuclear translocation and the subsequent upregulation of the ferroptosis-related genes ACSL4 and CHAC1, driving lipid peroxidation and cell death. Although our results indicate that YAP promotes ferroptosis in melanoma, the role of the Hippo-YAP axis in ferroptosis appears to be highly tumor type specific. For example, Xiao *et al.* 48 reported that in prostate cancer, tumor-derived EVs carrying miR-181a-5p suppress Hippo signaling in macrophages, promoting M2 polarization and ferroptosis resistance—a stark contrast to our findings in melanoma. These differences underscore the context-dependent role of Hippo-YAP signaling in ferroptosis regulation, suggesting that therapeutic strategies targeting this pathway, such as iEV-150, must be tailored to specific cancer types to maximize their efficacy.

In addition to direct tumor suppression, ferroptosis actively reshapes the tumor immune microenvironment (TME), particularly by regulating CD8+ T-cell function 49-52. Our study revealed that iEV-150 significantly increased CD8+ T-cell infiltration and granzyme B expression in tumor-bearing mice, suggesting improved antitumor immunity. Coculture experiments with iEV-150-treated A375 cells and CD8+ T cells further revealed enhanced T-cell cytotoxicity, marked by elevated TNF-α, IFN-γ, and Granzyme B levels, along with reduced levels of the exhaustion markers PD-1 and LAG-3. Notably, inhibiting the ferroptosis regulators CHAC1 and ACSL4 in A375 cells reversed these effects, highlighting ferroptosis as a critical mediator of T-cell activation, potentially through the release of immunogenic signals from dying tumor cells. This finding aligns with findings in colorectal cancer, where APOL3-induced ferroptosis increases CD8+ T-cell infiltration and effector function 53, and with the findings of Liao *et al.*, who reported that CD8+ T-cell-derived IFN-γ upregulates ACSL4 to increase ferroptosis and antitumor activity in melanoma 31. Similarly, Wang *et al.* reported that immunotherapy-activated CD8+ T cells enhance tumor ferroptosis via IFN-γ-mediated downregulation of SLC3A2 and SLC7A11, further supporting the interplay between ferroptosis and immune activation 54. These findings underscore the dual role of ferroptosis in modulating tumor immunity and emphasize the need to understand its regulatory mechanisms to design effective therapies that enhance antitumor responses while minimizing resistance 55.

Despite its promise, the translation of iEV-150 into clinical applications faces several challenges. Although iRGD modification enhances melanoma targeting, nonspecific accumulation in the liver and spleen persists. Strategies such as PEGylation 56 or functionalization with tumor-specific ligands (e.g., PD-L1-targeting peptides 57-59) could improve tumor specificity and circulation time, whereas immune-evasive coatings, such as macrophage-derived membranes, may reduce clearance by the mononuclear phagocyte system (MPS) and increase bioavailability 60-62. Additionally, the synergy between iEV-150 and immune checkpoint blockade (ICB) therapy offers a compelling avenue for overcoming ICB resistance in melanoma. Unlike prior studies focused solely on miRNA delivery, our findings demonstrate that iEV-150 enhances the efficacy of anti-PD-1 and anti-LAG3 therapies, suggesting a rational combination approach for clinical exploration. Future studies should investigate whether iEV-150 can further synergize with agents such as TGF-β inhibitors 63 or STING agonists 64, 65 to maximize antitumor immunity.

## Conclusions

In conclusion, iEV-150 is a novel biotherapeutic platform that integrates miRNA loading, iRGD-mediated tumor targeting, ferroptosis induction, and immune modulation through enhanced CD8+ T-cell activity. Tumor cell ferroptosis is induced while antitumor immunity is increased, suggesting a dual-action approach to combat tumor growth and immune evasion in melanoma. Future advancements in EV engineering, targeting strategies, and combination therapies, such as with immune checkpoint inhibitors, will further enhance its clinical potential. The safety profile, biodistribution, and broader effects on the tumor microenvironment of iEV-150 will need to be better understood to make progress toward its clinical application, potentially establishing a new paradigm for RNA-based immunotherapies in melanoma and other cancers.

## Materials and Methods

### Clinical samples and tissue microarray

Peripheral blood samples were collected from 16 melanoma patients who had not received prior chemotherapy or radiotherapy at the Cancer Hospital, Chinese Academy of Medical Sciences (Approval No.: NCC2025C-088). In accordance with the Declaration of Helsinki, informed consent was obtained from all participants. The blood samples were centrifuged at 3,000 × g for 10 min at 4 °C to separate the plasma supernatants, with the procedure completed within 4 h. Subsequently, the plasma samples underwent further centrifugation at 16,000 × g for 10 min at 4 °C and were stored at -80 °C for subsequent exosomal extraction. The melanoma tissue microarray (AF-MelalSur2401) was purchased from Hunan Aifang Biotechnology Co., Ltd., and included 70 paired samples of adjacent normal tissues and melanoma tissues. The researchers performed *in situ* hybridization, immunohistochemistry (IHC), and Prussian blue staining on the TMA. The results were independently evaluated by two professional pathologists, as follows: the score was calculated as the proportion of tumor cells (SPARC for stromal cells) × staining intensity. The proportion was graded as 0 (no stained cells), 1 (< 10%), 2 (10%-25%), 3 (26%-49%), or 4 (≥ 50%). The staining intensity was graded as 0 (negative), 1 (light yellow), 2 (yellow-brown), or 3 (brown). Samples with total scores of 0-4 were classified as low miR-150-3p expression, while scores ≥ 6 indicated high miR-150-3p expression. The study utilizing the tissue microarray was approved by the Life Sciences Ethics Committee of Changsha Yaxiang Biotechnology Co., Ltd. (Approval No.: Csyayj2024053).

### Cell lines and cell culture

Human epidermal melanocyte cell line PIG1 and human melanoma cell lines A375 and A875 were obtained from the American Type Culture Collection (ATCC, Manassas, VA, USA). The murine melanoma cell line B16-F10 was obtained from Pricella (Wuhan, China). PIG1, A375, and B16-F10 cells were cultured in RPMI-1640 medium (VivaCell, Shanghai, China) supplemented with 10% fetal bovine serum (FBS, Gibco, Grand Island, NY, USA) and 1% penicillin-streptomycin (Beyotime, Shanghai, China). A875 cells were cultured in MEM medium (VivaCell, Shanghai, China) containing 10% FBS and 1% penicillin-streptomycin. Cells were maintained at 37 °C in a humidified incubator with 5% CO₂.

### miRNA sequencing and mRNA sequencing

Total RNA was extracted using TRIzol reagent (TaKaRa, Tokyo, Japan) and purified with the RNeasy Mini Kit (QIAGEN, Dusseldorf, Germany), following the manufacturers' instructions. RNA quality and concentration were measured using a Nanodrop ND-1000 spectrophotometer (Nanodrop Technologies). miRNA sequencing of A875 cells and derived EVs, as well as mRNA sequencing of A875 cells co-cultured with iEV-NC or iEV-150, were conducted using the NovaSeq 6000 platform (Illumina, San Diego, CA, USA). For miRNA analysis, replicates were averaged, and miRNAs with expression intensities ≥ 30 in all samples were selected for normalization. Median normalization was applied. Differential expression analysis was performed using DESeq2, and hierarchical clustering was used to visualize differentially expressed genes (DEGs). Heat map analysis included all DEGs. Kyoto Encyclopedia of Genes and Genomes (KEGG) enrichment analysis was conducted based on a hypergeometric distribution algorithm to identify significantly enriched pathways.

### EV isolation and purification

EVs were isolated from cell culture supernatants through sequential centrifugation at 4 °C (300 × g for 10 min, followed by 200 × g for 10 min), and filtration through a 0.22 µm membrane (Millipore, USA). The supernatant was further centrifuged at 10,000 × g for 30 min and concentrated using a 100 kDa centrifugal filter (Millipore, USA). EVs were then purified by ultracentrifugation at 120,000 × g for 90 min, washed with phosphate-buffered saline (PBS), subjected to a second ultracentrifugation at 120,000 × g, and resuspended in 200 µL PBS. Plasma EVs were extracted using a size exclusion chromatography (SEC) kit (Exosupur, Echo Biotech, China) and concentrated with a 100 kDa centrifugal filter. Freeze-thaw cycles were strictly avoided. EV characterization was performed via nanoparticle tracking analysis (NTA), transmission electron microscopy (TEM), and Western blot analysis for EV-specific markers.

### Nanoparticle tracking analysis (NTA)

EV samples were diluted in 500 µL particle-free PBS (filtered through 0.02 µm filter) and analyzed using a NanoSight NS500 system (Malvern Instruments, Amesbury, UK). Focal length and camera parameters were optimized according to manufacturer guidelines. Five independent videos (45 s each) were recorded, and particle concentrations and sizes were analyzed using NTA software (version 3.4).

### Transmission electron microscopy (TEM)

EV samples suspended in particle-free PBS (0.02 µm filtered) were placed onto carbon-coated copper grids (200 mesh) and incubated at room temperature for 5 min. Excess liquid was removed, and grids were negatively stained with uranyl acetate for 10 s. After removing excess stain, grids were air-dried for 30 min and imaged using a transmission electron microscope (JEOL, USA). For mitochondrial morphology assessment, melanoma cells (A375 and A875) were pelleted by centrifugation (3,000 rpm for 20 min), fixed in cacodylate-buffered 1% osmium tetroxide, dehydrated through graded ethanol series, embedded in Poly/Bed 812 resin, and subsequently imaged via TEM.

### Liquid chromatography-mass spectrometry (LC-MS/MS)

Protein bands obtained from miR-150-3p RNA pull-down assays were analyzed by LC-MS/MS using a Q Exactive mass spectrometer coupled with a Dionex Ultimate 3000 RSLCnano liquid chromatography system (Thermo Scientific). Samples from three biological replicates were individually processed and subsequently pooled to enhance detection sensitivity. Samples were digested overnight at 37 °C with 20 ng/µl trypsin in 25 mM NH₄HCO₃. Peptides were purified and dissolved in sample solvent (0.1% formic acid, 2% acetonitrile) before analysis. Peptide separation was performed on an Acclaim PepMap RSLC C18 column (300 µm ID × 5 mm, 5 µm, 100 A, Thermo, 160454). Key MS2 parameters included a maximum ion injection time (IT) of 60 ms and a normalized collision energy (NCE) of 27. Raw MS data were converted to MGF format using MM File Conversion software and analyzed via the MASCOT search engine against the UniProt database.

### Construction of engineered EVs

Lentiviral vectors, including pLV-Puro, miR-150-3p overexpression lentivirus, and ANXA2 overexpression lentivirus, were constructed by Fenghui Biotechnology Co., Ltd. (Hunan, China). The miR-150-3p overexpression lentivirus was transfected into HEK-293T cells at an MOI of 5. Following infection, the cells were selected with 5 μg/mL puromycin for 14 d and then maintained in puromycin-free medium, generating a stable HEK-293T cell line overexpressing miR-150-3p. qRT-PCR analysis confirmed the upregulation of miR-150-3p in both cells and EVs. Since ANXA2 overexpression enhances the incorporation of miR-150-3p into EVs, the ANXA2 overexpression lentivirus was subsequently transfected into the miR-150-3p-overexpressing cells, resulting in a stable HEK-293T cell line co-overexpressing miR-150-3p and ANXA2. qRT-PCR analysis further validated the increased miR-150-3p levels in EVs. To engineer EVs, the ExoBrooch-iRGD kit (ECHO BIOTECH, Beijing, China) was used to conjugate Chol-PEG-iRGD (a peptide, CRGDKGPDC, targeting integrins αvβ3 and αvβ5) to the miR-150-3p-loaded EVs. Briefly, 50 μg of Chol-PEG-iRGD was incubated with 10^7^ particles EVs at 37 °C for 5 h. The mixture was then subjected to three rounds of 30-minute centrifugation at 4000 rpm to obtain the engineered EVs (iEV-150).

### EV labeling and visualization

EVs were labeled using PKH67 membrane dye (Sigma, USA). Briefly, EVs (2 × 10^5^ particles in 20 µL PBS) were incubated with PKH67 dye diluted in diluent C for 5 min, washed by ultracentrifugation, and resuspended in PBS. Recipient cells (2 × 10^5^) were incubated with labeled EVs (5 µg) for 24 h, fixed with 4% paraformaldehyde, permeabilized with 0.5% Triton X-100, stained with 500 nM Phalloidin, counterstained with DAPI, and visualized by fluorescence microscopy.

### Biodistribution of EVs *in vivo*

C57BL/6 mice bearing subcutaneous B16-F10 tumors (5 × 10^6^ cells injected) were intravenously injected with 5 × 10^10^ particles DIR-labeled EV-150 or iEV-150 particles. Whole-body fluorescence imaging was performed at 3, 6, and 9 h post-injection using IVIS. At 9 h, organs were harvested for *ex vivo* imaging. Tumors were fixed, sectioned (20 µm), stained with DAPI, and analyzed using confocal fluorescence microscopy.

### RNA immunoprecipitation (RIP) assay

RIP assays were conducted using the Magna RIP RNA-Binding Protein Immunoprecipitation Kit (Millipore, MA, USA). Briefly, cell lysates supplemented with protease and RNase inhibitors were incubated overnight at 4 °C with anti-AGO2 antibody-coupled magnetic beads. Bead-bound RNA-protein complexes were washed, digested with Proteinase K, and the isolated RNA was analyzed by qRT-PCR.

### Isolation of RISC-associated RNA

Cells overexpressing miR-150-3p or NC were fixed with 1% formaldehyde, lysed in NETN buffer, and incubated with Dynabeads Protein A (Invitrogen, USA) coupled with anti-Pan-Ago or IgG antibodies (clone 2A8, Millipore, USA). Immunoprecipitated RNA was purified by phenol-chloroform extraction and ethanol precipitation, followed by DNase I treatment.

### Biotin RNA pulldown assay

Cells were lysed in RIP buffer containing protease and RNase inhibitors (Invitrogen). Cell lysates were pre-cleared, then incubated at 4 °C for 4 h with biotin-labeled RNA probes immobilized on streptavidin-coated magnetic beads (Invitrogen, USA). Beads were extensively washed, and bound proteins/RNA were eluted in Laemmli buffer for Western blot analysis.

### Co-immunoprecipitation (Co-IP) assay

Cells transfected with indicated plasmids were lysed in NP40 buffer with protease inhibitors. Supernatants were incubated overnight with primary antibodies at 4 °C, followed by Protein A/G-agarose beads (Santa Cruz Biotechnology) precipitation. Bead-bound complexes were washed, resuspended in SDS buffer, and analyzed by Western blot analysis.

### Electrophoretic mobility shift assay (EMSA)

EMSA was performed using the EMSA Kit (Thermo Fisher Scientific) according to the manufacturer's instructions. Briefly, biotin-labeled DNA or RNA probes and unlabeled competitor probes were prepared in 5 × TBE buffer. Binding reactions were performed by incubating the probes with protein extracts at room temperature for 20 min. Samples were then resolved by electrophoresis on a pre-electrophoresed non-denaturing polyacrylamide gel. After electrophoresis, samples were transferred onto membranes, crosslinked by UV irradiation, blocked, and incubated with stabilized streptavidin-HRP conjugate. Signal detection was performed by chemiluminescence.

### Dual-luciferase reporter assay

Luciferase reporter plasmids were co-transfected into A375 and A875 cells along with Renilla luciferase plasmids. After 48 h incubation, cells were lysed using passive lysis buffer, and luciferase activities were measured immediately using a Dual-Luciferase Reporter Assay System (Promega, WI) on a microplate reader.

### RNA extraction and quantitative real-time PCR (qRT-PCR)

Total RNA was extracted from cells and EVs using TRIzol reagent (Invitrogen, USA). miRNA and mRNA cDNA were synthesized using the miR-X miRNA First-Strand Synthesis Kit (TaKaRa, Tokyo, Japan) and PrimeScript™ RT reagent Kit with gDNA Eraser (TaKaRa, Tokyo, Japan), respectively. Cel-miR-39 (TIANGEN, China) was added to EV pellets as an exogenous reference prior to miRNA extraction. qRT-PCR was performed with GoTaq® Real-Time PCR System (Promega) on an Applied Biosystems 7500 instrument. Gene expression was analyzed by the 2^-ΔΔCT^ method, using GAPDH (mRNA), U6 (cellular miRNA and EVs from cell culture), or miR-191-5p (plasma EV miRNA) for normalization. Primer sequences are provided in Table S2.

### Western blot analysis

Whole-cell lysates were obtained by centrifugation at 12,000 × g for 15 min after lysis. Protein concentrations were measured using a BCA assay. Proteins were separated by 10% SDS-PAGE, transferred to polyvinylidene fluoride membranes (Millipore, MA, USA), blocked in 5% BSA, and incubated with primary antibodies overnight at 4 °C. Membranes were washed, incubated with HRP-conjugated secondary antibodies, and signals were visualized using Clarity Western ECL Substrate (Bio-Rad, CA, USA). Antibody details are listed in Table S3.

### Lentiviral transfection

Lentiviruses (pLV-Puro, miR-150-3p-overexpression, NF2-overexpression) were purchased from Fenghui Biotechnology (Hunan, China). Lentiviral vectors were based on a third-generation system with a CMV promoter and GFP tag. A375 and A875 cells were infected at MOI 10 and HEK-293T at MOI 5, selected with puromycin (5 µg/mL) for 14 d, and maintained without puromycin thereafter. Puromycin selection was repeated every 30 d for sustained expression.

### Plasmid construction and cell transfection

miRNA inhibitors, sgRNAs targeting ANXA2 and YAP, shRNAs targeting ACSL4 and CHAC1, and overexpression plasmids for ANXA2 and YAP were synthesized by GenePharma. For transient transfection, plasmids (2 μg/mL) and shRNAs (50 nM) were transfected using the Lipofectamine™ 3000 kit (Thermo Fisher Scientific, Waltham, USA) according to the manufacturer's instructions. Sequences are provided in Table S4.

### Cell counting kit-8 (CCK-8), and 5-ethynyl-20-deoxyuridine (EdU)

Melanoma cells were seeded into 6-well plates, transfected, and incubated for 48 h. EdU assays were conducted using an EdU Cell Proliferation Assay Kit (RiboBio, Guangzhou, China). For CCK-8 assays (Beyotime), cells (1 × 10³ cells/well) were seeded into 96-well plates, treated at designated times, incubated with CCK-8 reagent for 2 h, and absorbance measured at 450 nm.

### Colony formation assay

Cells (1,000 per well) were seeded in 6-well plates, incubated for 14 d, fixed with 4% paraformaldehyde, and stained with 0.5% crystal violet (Beyotime). Colonies were subsequently quantified.

### Tumorisphere formation

A875 (5 × 10³) and A375 (2 × 10³) cells were cultured in low-attachment dishes (Corning Costar, USA) using MammoCult™ Human Medium (STEMCELL Technologies, USA) at 37 °C with 5% CO₂ for 14 d. Tumorispheres were quantified from five random fields.

### Animal models and experimental procedures

C57BL/6 mice (4-6 weeks old, SpePharm, Beijing, China) were housed under SPF conditions at the Laboratory Animal Center of Inner Mongolia University. For subcutaneous tumor models, 5 × 10⁵ B16F10 cells were injected into C57BL/6 mice; from day 7, iEV-NC or iEV-150 (5 × 10¹⁰ particles) was administered intravenously every three days, with tumors harvested on day 21. For metastasis models, 5 × 10⁵ B16F10-luc cells were injected intravenously into BALB/c nude mice; iEV treatments began on day 5, with fluorescence imaging and lung analysis on day 21. For immunotherapy, C57BL/6 mice with B16F10 subcutaneous tumors received anti-PD-1, anti-LAG-3, isotype control IgG, or EVs (as described) every three days from day 3. All procedures were approved by the Institutional Animal Care and Use Committee (IMU-mouse-2022-067).

### RNA fluorescence *in situ* hybridization (FISH)

Cells were embedded into paraffin sections, permeabilized, and hybridized overnight at 37 °C with fluorescence-labeled miR-150-3p probes. Sections were counterstained with DAPI and visualized using confocal laser microscopy.

### TUNEL staining

TUNEL staining was performed using a TUNEL assay kit (Roche, 11684817910) per manufacturer guidelines. Paraffin-embedded sections (3 µm) were deparaffinized, rehydrated, and treated with proteinase K (37 °C, 20 min). Sections were incubated with TUNEL reaction mixture at 37 °C for 2 h, counterstained with DAPI (37 °C, 10 min), mounted using anti-fade medium, and imaged using a Nikon fluorescence microscope. TUNEL-positive cells were quantified manually.

### Prussian blue and DAB staining

Paraffin sections were deparaffinized, rehydrated, and stained in equal parts of 2% potassium ferrocyanide and 2% hydrochloric acid for 30 min. Sections were counterstained with hematoxylin for 1 min, differentiated, rehydrated, and mounted in neutral resin for microscopic observation.

### Masson staining

Masson staining was performed on mouse tumor sections using a Masson staining kit (Servicebio, China). Sections were deparaffinized, rehydrated, and immersed overnight in Masson A solution. Subsequently, sections were stained with a 1:1 mixture of Masson B and C solutions (1 min), briefly differentiated, stained with Masson D solution (6 min), rinsed, stained with Masson E (1 min), and immediately treated with Masson F (2-30 s). Sections were differentiated, rinsed in 1% acetic acid, mounted, and analyzed under a microscope.

### Ferroptosis index assay

Intracellular total iron content and ferrous iron levels were quantified using colorimetric assay kits (E-BC-K880-M and E-BC-K881-M; Elabscience, Wuhan, China). Cells (2 × 10⁵ per well) were harvested after 24 h treatment, lysed, centrifuged (15,000 × g, 10 min), and supernatants incubated with chromogenic solutions at 37 °C (total iron: 40 min; ferrous iron: 10 min). Absorbance was measured at 593 nm.

MDA levels were assessed using a colorimetric kit (E-BC-K028-M; Elabscience). Treated cells were lysed and centrifuged, and 0.1 mL of each supernatant was incubated with 1 mL working solution at 100 °C for 40 min. After cooling and centrifugation (15,000 × g, 10 min), absorbance was measured at 532 nm.

Lipid ROS levels were assessed by staining cells with 10 μM C11-BODIPY (Thermo Fisher, Cat. #D3861) for 30 min at 37 °C, followed by PBS washing and flow cytometric analysis of fluorescence (excitation/emission: 484/510 nm).

Mitochondrial superoxide levels were measured by staining cells with 10 μM MitoSOX™ Red (Invitrogen) for 10 min at 37 °C, followed by PBS washing and flow cytometric analysis (excitation/emission: 510/580 nm).

GSH and GSSG levels were quantified using a GSH/GSSG Detection Assay Kit (Abcam, ab138881) according to the manufacturer's instructions.

Mitochondrial membrane potential was assessed by incubating cells with JC-1 staining solution (Beyotime, C2003S) at 37 °C for 20 min, followed by two PBS washes and analysis by fluorescence microscopy or flow cytometry.

### *In vitro* CD8+ T cell preparation and activation

Peripheral blood mononuclear cells (PBMCs) from 16 melanoma patients were isolated using Ficoll® Paque Plus (Sigma, USA). CD8⁺ T cells were separated using Dynabeads™ CD8 Positive Isolation Kit (Invitrogen), activated with anti-CD3/anti-CD28 beads (Dynabeads CD3/CD28, Invitrogen) and recombinant IL-2 (Abcam, UK).

### CFSE

CellTrace™ CFSE Cell Proliferation Kit (C34554, Invitrogen, USA) was used to assess CD8+ T cell proliferation. CD8+ T cells co-cultured with A375 cells were collected and resuspended in PBS. CFSE working solution was added to a final concentration of 5 μM, followed by incubation in the dark. The staining reaction was terminated by adding 10-fold diluted FBS. CFSE fluorescence intensity was analyzed using a flow cytometer, and a CFSE fluorescence histogram was generated with FlowJo for data visualization.

### Flow cytometry

To evaluate CD8⁺ T cell cytokine secretion, CD8⁺ T cells isolated from PBMCs were cultured for 3 d post-treatment. After Fc receptor blocking and GolgiStop treatment, cells were stained with antibodies against CD45, CD3, CD8, PD-1, and LAG-3 (Invitrogen) at 4 °C for 30 min. Intracellular staining for IFN-γ, Granzyme B, Perforin, and TNF-α (Invitrogen) was performed using the FIX & PERM™ Cell Permeabilization Kit (Invitrogen). Samples were analyzed on a BD FACSVerse Flow Cytometer.

For tumor immune cell profiling, mouse tumor tissue was minced, digested with 0.5 mg/mL collagenase D and 0.25 mg/mL DNase I (Roche; cat. no. COLLD-RO) at 37 °C for 1 h, filtered through a 70 μm mesh, and lysed with ACK buffer. Single-cell suspensions were stained with antibodies against CD3, CD8, CD4, F4/80, CD11b, NK1.1, and CD45 (Invitrogen) for flow cytometry.

Cell cycle analysis was performed by incubating A375 and A875 cells with PI/RNase A solution (Sigma-Aldrich, St. Louis, MO, USA) and analyzing via flow cytometry. Apoptosis was assessed using the Annexin V-FITC Apoptosis Detection Kit (Invitrogen), with cells stained with Annexin V-FITC and propidium iodide.

### Enzyme-linked immunosorbent assay (ELISA)

After 48 h of co-culture with A375 cells, culture supernatants from CD8⁺ T cells were collected. Levels of IFN-γ, TNF-α, Granzyme B, Perforin, PD-1, and LAG-3 were quantified using ELISA kits (Elabscience) in accordance with the manufacturer's instructions. Absorbance at 450 nm was measured using a microplate reader.

### CD8⁺ T cell and A375 co-culture

CD8⁺ T cells and pre-treated A375 cells were co-cultured in a Transwell system (Corning, pore size: 0.4 μm) at a ratio of 1:3. CD8⁺ T cells were seeded in the lower chamber, and A375 cells in the upper chamber. After 48 h, the upper chamber was removed, and CD8⁺ T cells were collected for further analysis.

### Immunohistochemistry and hematoxylin and eosin (H&E) staining

Paraffin-embedded tissue sections were deparaffinized, rehydrated, and subjected to heat-mediated antigen retrieval in 10 mM sodium citrate buffer (pH 6.0). Endogenous peroxidase activity was blocked with 3% H₂O₂ for 10 min. Sections were blocked with 5% BSA and incubated with primary antibodies overnight at 4 °C, followed by secondary antibody incubation at 37 °C for 30 min. Visualization was performed using 3,3′-diaminobenzidine (DAB), and slides were examined microscopically. For histopathological evaluation, tumor and lung tissues were stained with H&E. Briefly, sections were dewaxed in xylene, rehydrated through graded ethanol, stained with hematoxylin for 5 min at room temperature, rinsed, differentiated in 1% acid alcohol for several seconds, washed in running water for 30-60 min, counterstained with eosin for 2 min, and rinsed for final observation. Detailed antibody information is provided in Table S3.

### Immunofluorescence assay

Cells were seeded in confocal dishes and fixed with 4% paraformaldehyde (PFA) for 10 min the following day. After blocking with blocking buffer (Beyotime, Shanghai, China) for 2 h, cells were incubated with primary antibodies overnight at 4 °C. The next day, cells were washed and incubated with fluorophore-conjugated secondary antibodies (1:200) in the dark for 2 h. After washing, nuclei were stained with DAPI (Beyotime), and samples were stored at 4 °C in the dark until imaging. Images were acquired using a confocal microscope. Antibodies used are listed in Table S3.

### Bioinformatics

Single-cell RNA sequencing data from melanoma patients (GSE148190, GSE123139, GSE179373) were analyzed using the Seurat package (v4.3.0) in RStudio. After normalization, the top 2000 variable genes were identified with FindVariableFeatures, followed by PCA. Clustering was performed using FindNeighbors (dims = 1:15) and FindClusters with a resolution of 0.5. Immune cell types were annotated based on known marker genes. Bulk transcriptomic data from TCGA and GTEx were also used to evaluate candidate miR-150-3p target gene expression in SKCM samples. Differential miRNA expression between melanoma cells and EVs was analyzed using datasets GSE125030 and GSE35387, processed with the limma package. Criteria for differential expression were p < 0.05 and |log₂FC| > 2.

### Statistical analysis

Statistical analyses were performed using SPSS 19.0 (IBM, NY, USA) and GraphPad Prism 8.0 (GraphPad Software, CA, USA). Comparisons between two groups were made using two-tailed Student's t-tests. Spearman correlation analysis was conducted to evaluate the association between miR-150-3p and target gene expression. Kaplan-Meier survival analysis was used for overall and progression-free survival. All experiments were independently performed at least three times. Data are expressed as mean ± SD. Statistical significance was defined as follows: n.s. = not significant; *p < 0.05, **p < 0.01, ***p < 0.001. Data collection from human melanoma tissues, plasma samples, animal models, and other biological materials was performed in a blinded manner, and all data were included in the final analysis.

## Supplementary Material

Supplementary figures and tables.
