# Supplementary material for: Engineered nanovesicle platform simultaneously triggers YAP-dependent ferroptosis and reprograms T-cell immunity through miR-150-3p codelivery in melanoma microenvironment
Source: Theranostics. 2025 Jul 25;15(16):8377–403. doi: 10.7150/thno.115860 (PMC12374587; doi:10.7150/thno.115860)
Supplement: Supplementary file 1 — Supplementary figures and tables. [file thnov15p8377s1.pdf]

Supplemental information

Engineered Nanovesicle Platform Simultaneously Triggers YAP-dependent Ferroptosis and Reprograms T-Cell Immunity through miR-150-3p Codelivery in Melanoma Microenvironment

Jiemin Wang[1†], Zhenguo Zhao[2†], Haopeng Yang[1], Ruixuan Wang[1], Shu Wang[1], Jiale Yu[1], Yujia Wang[1], Ruihua Liu[1], Yani Chen[1], Yueshi Liu[1], Kesong Shi[1], Pengyong Han[1], Miao Liu[1], Jing Miao[1], Xiaoyang Li[2], Xiangnan Li[1], Haiquan Yu[1*]

[1] State Key Laboratory of Reproductive Regulation and Breeding of Grassland Livestock, School of Life Sciences, Inner Mongolia University, Hohhot 010020, Inner Mongolia, China.
[2] Department of Orthopaedics, National Cancer Center/National Clinical Research Center for Cancer/Cancer Hospital, Chinese Academy of Medical Sciences and Peking Union Medical College, 100021, Beijing, China.

[†]Jiemin Wang and Zhenguo Zhao contributed equally to this study.

*Corresponding Author: Haiquan Yu

State Key Laboratory of Reproductive Regulation and Breeding of Grassland Livestock, School of Life Sciences, Inner Mongolia University, Hohhot 010020, Inner Mongolia, China; Email: hyu@imu.edu.cn

1. Supplemental figure 1-14

2. Supplemental Table 1-4

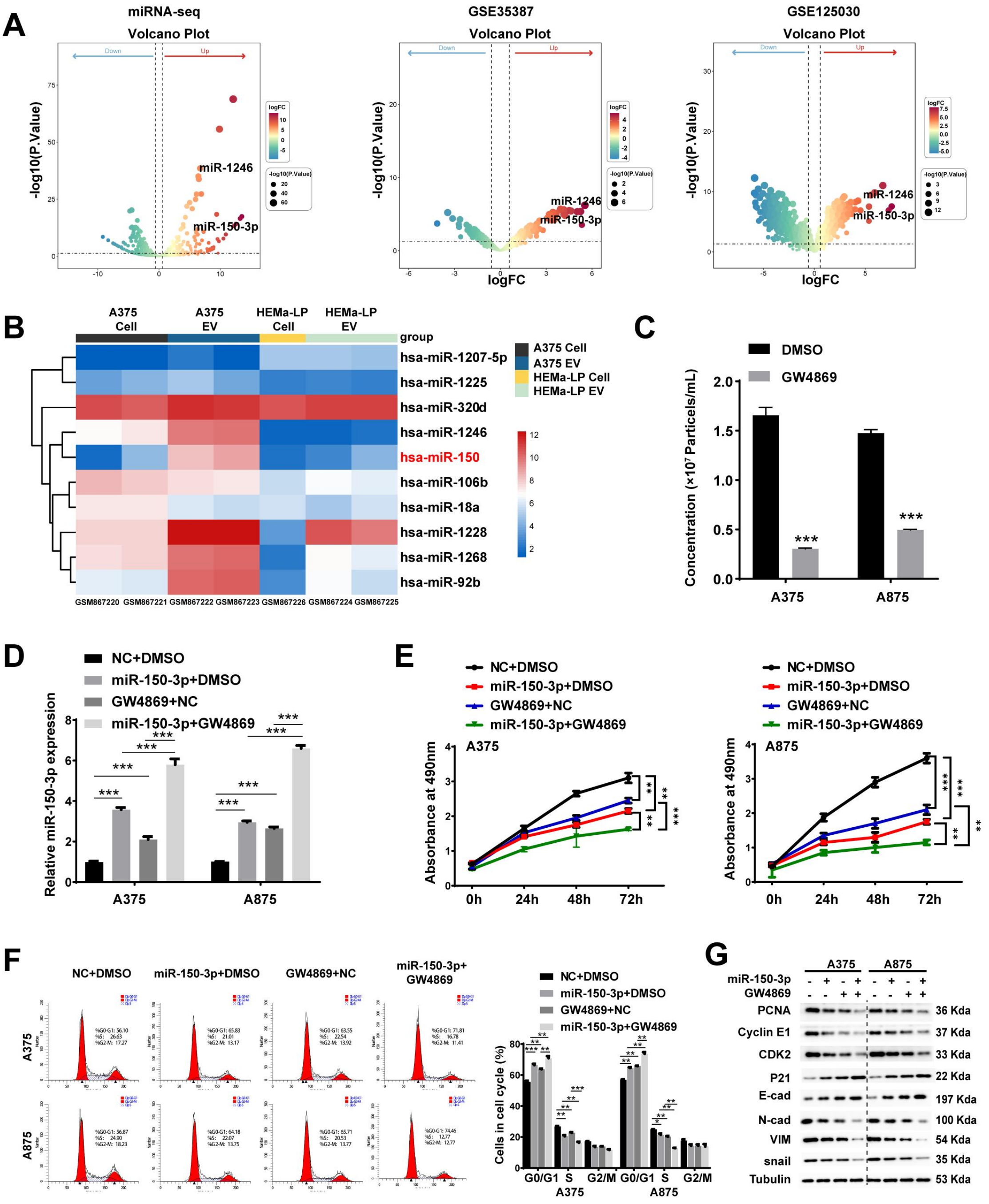

Supplementary Figure 1：Inhibition of miR-150-3p loading into extracellular vesicles suppresses melanoma cell proliferation and EMT. A The volcano plot illustrates the expression distribution of differentially expressed miRNAs across the three datasets. **B** A four-group heatmap showing the expression of miR-150-3p in GSE35387 . **C** EV production after treatment with 10 μM GW4869. **D** The intracellular expression level of miR-150-3p in GW4869-treated cells was assessed using qRT-PCR. **E** Cell proliferation in A375 and A875 cells after overexpression of miR-150-3p and GW4869 treatment was assessed using the CCK-8 assay. **F** The effect of miR-150-3p overexpression and GW4869 treatment on the cell cycle of melanoma cells was analyzed using flow cytometry. **G** Western blot analysis was performed to assess the

levels of cell proliferation and EMT-related proteins. Mean ± SD, *p < 0.05; **p < 0.01; ***p < 0.001.

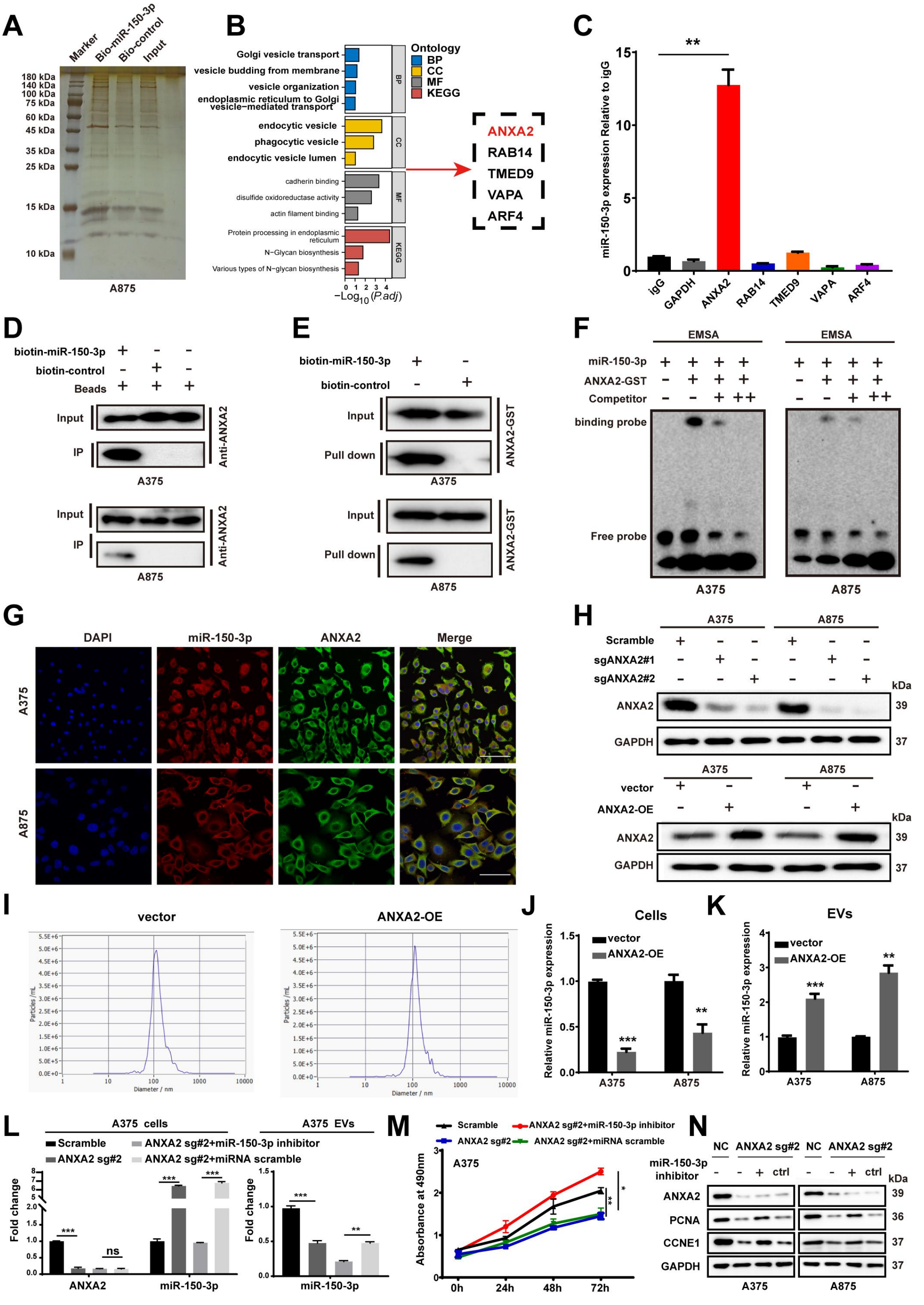

Supplementary Figure 2 Sorting of miR-150-3p from cell to EVs through Annexin A2. **A** Biotin-miR-150-3p complex was pulled down from whole-cell extracts using

streptavidin beads, followed by electrophoresis and silver staining for analysis. **B** GO and KEGG analyze the function of RBPs. **C** qRT-PCR detection of miR-150-3p in IgG, GAPDH, ANXA2, RAB14, TMED9, VAPA and ARF4 immunoprecipitants of A875 lysate. **D** RNA-protein pull-down using a biotin-labeled miR-150-3p probe or control probe with melanoma cell lysate. **E** RNA pull-down assays were conducted with biotin-miR-150-3p or biotin-control bound to streptavidin-conjugated beads and incubated with the indicated GST-tagged-ANXA2 purified from E. coli. The results were assessed by Western blot analysis. **F** EMSA analysis of the interactions between biotin-miR-150-3p (2 nM) and recombinant GST-tagged-ANXA2 . Signals were revealed by Streptavidin-HRP. **G** Immunofluorescence analysis of miR-150-3p and ANXA2 localization in A375 and A875 melanoma cells. Scale bar represents 50 µm. **H** Top: Western blot analysis of ANXA2 in melanoma cells with or without CRISPR/Cas9-mediated ANXA2 knockout. Bottom: Western blot analysis of ANXA2 in melanoma cells transfected with ANXA2 overexpression vector or empty vector. **I** NTA analysis of EV concentration from A375 cells after ANXA2 overexpression. **J, K** qRT-PCR analysis of miR-150-3p expression in melanoma cells (J) and EVs (K) after ANXA2 overexpression. **L** qPCR-quantification of miR-150-3p and ANXA2 expressed in A375 cells (left panel) and A375 EVs (right panel) after the cells were treated as indicated. **M** Detection of cell proliferation by CCK8 assay. **N** Western blot analysis showing the level of ANXA2, PCNA and CCNE1 in cell lysates treated as indicated. Mean ± SD, *p < 0.05; **p < 0.01; ***p < 0.001.

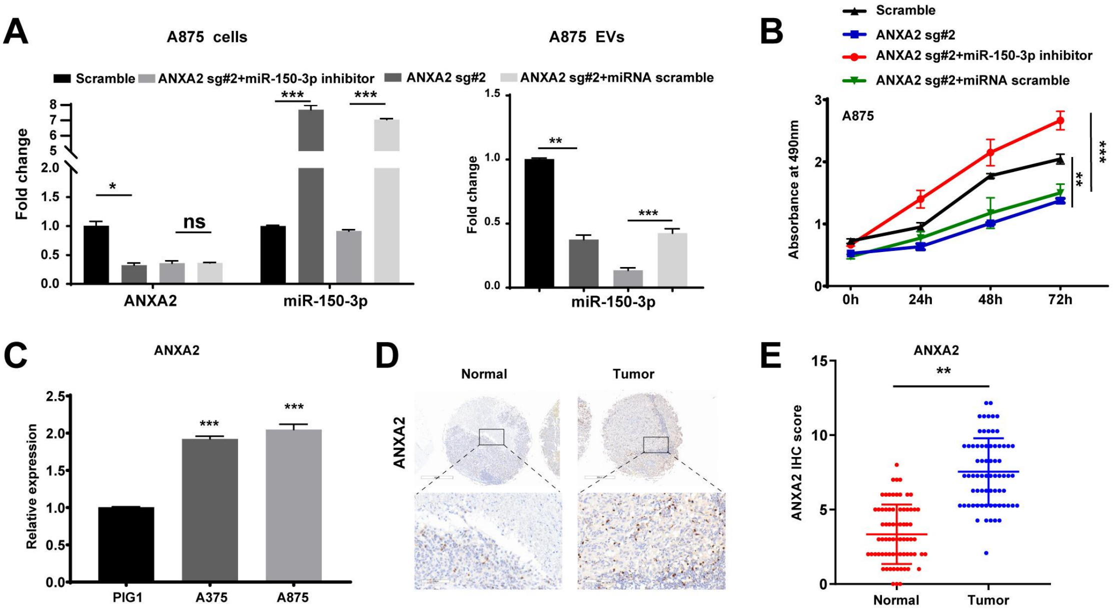

Supplementary Figure 3： ANXA2 regulates the incorporation of miR-150-3p into EVs. **A** qPCR-quantification of miR-150-3p and ANXA2 expressed in A385 cells (left panel) and A875 EVs (right panel) after the cells were treated as indicated. **B** Detection of cell proliferation by CCK8 assay. **C** qRT-PCR analysis of ANXA2 expression

in melanoma cell lines A375 and A875, as well as in normal melanocytes PIG1. **D, E** Representative picture of ANXA2 protein expression in melanoma tissue chip detected by IHC and quantification of ANXA2 protein was shown. Scale bar = 100 µm. Mean ± SD, *p < 0.05; **p < 0.01; ***p < 0.001.

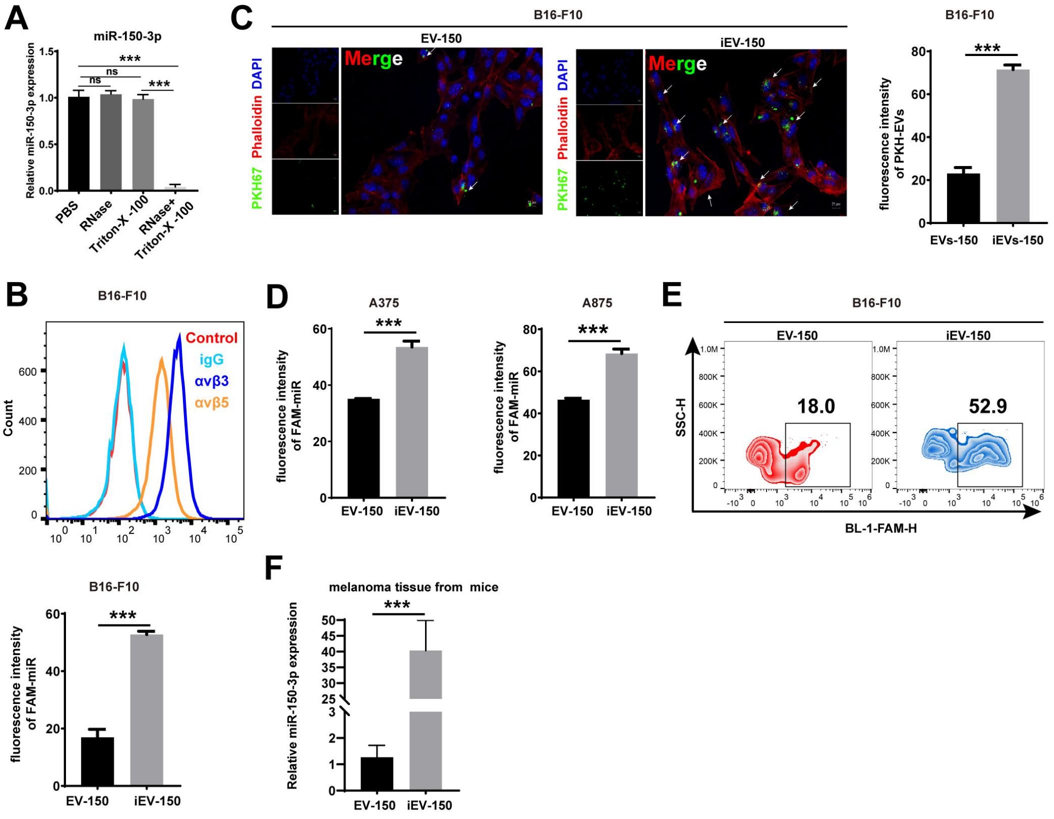

Supplementary Figure 4： iEV-150 with Targeting Peptide Increases Targeting of Melanoma Cells in Vivo and In Vitro. A Quantification of miR-150-3p in iEV-150 following treatment with PBS (control), RNase A, Triton X-100, combination of RNase A and Triton X-100 . U6 was used as the reference gene. **B** Flow cytometric analysis of surface integrin expression in B16-F10 melanoma cells. Histograms show staining with anti-αvβ3 (blue) and anti-αvβ5 (orange) antibodies, compared with isotype control (cyan, mouse IgG1) and unstained cells (red). The elevated signals indicate high expression levels of both integrins. **C** Laser scanning confocal imaging of B16-F10 cells uptake of PKH67 labeled EVs (scale bar = 100 µm) and analysis of fluorescence intensity of uptake. **D** Flow cytometry analysis of A375 and A875 cells for statistical analysis of FAM-labeled engineered iEV-150 and EV-150. **E** Flow cytometry analysis of B16-F10 cells uptake of FAM-labeled engineered iEV-150 and EV-150. **F** qRT-PCR analysis of miR-150-3p expression in melanoma tissues from C57BL/6 mice. Mean ± SD, *p < 0.05; **p < 0.01; ***p < 0.001.

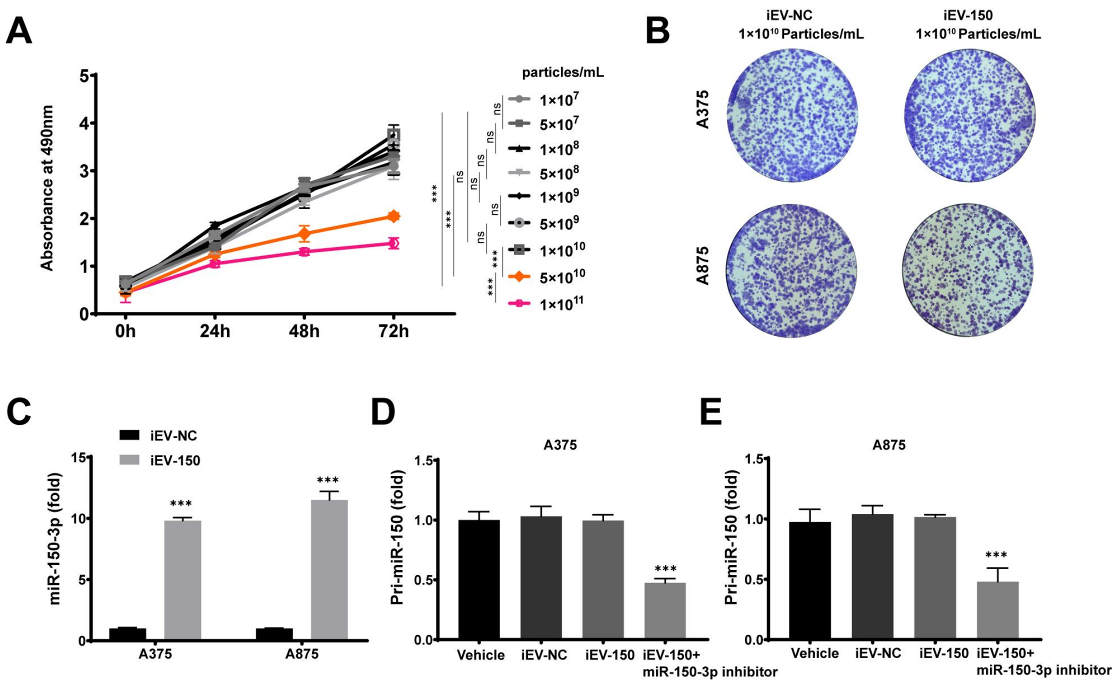

Supplementary Figure 5： Optimizing the concentration of engineered EVs to explore modulation of melanoma cell biological behavior. A CCK-8 assay of A375 cells treated with different concentrations of iEV-150. **B** Colony formation assay of A375 and A875 cells treated with iEV-150 (1×10^{10} particles/mL) and iEV-NC (1×10^{10} particles/mL). **C** The expression change of miR-150-3p after co-culturing A375 and A875 cells with iEV-NC or iEV-150 was detected by qRT-PCR. **D, E** qRT-PCR was performed to detect the expression level of pre-miR-150 in A375 and A875 cells after different treatments. Mean ± SD, *p < 0.05; **p < 0.01; ***p < 0.001.

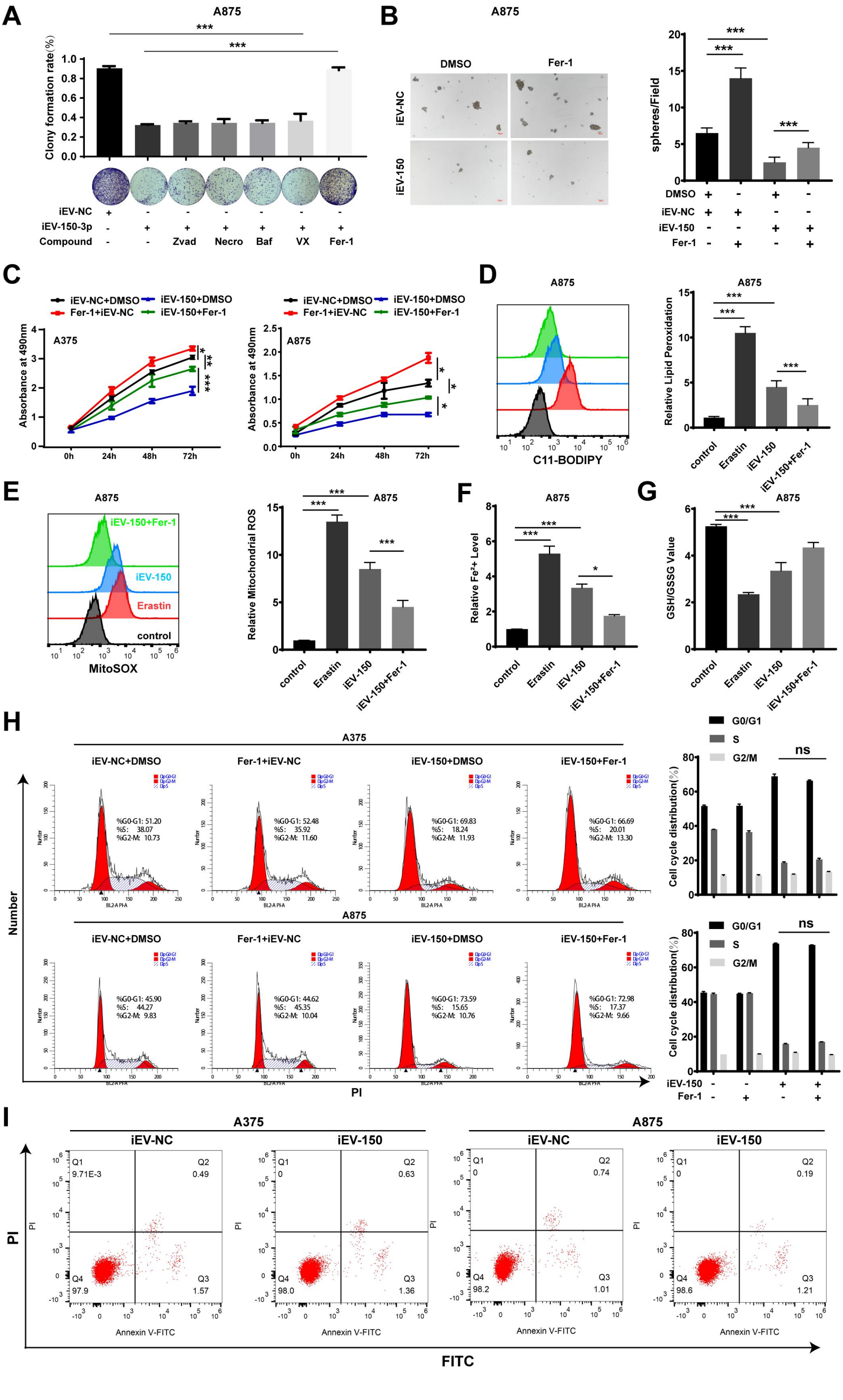
A
A875
Clony formation rate(%)
iEV-NC
iEV-150-3p
Compound
Zvad
Necro
Baf
VX
Fer-1
B
A875
DMSO
Fer-1
iEV-NC
iEV-150
spheres/Field
C
iEV-NC+DMSO
iEV-150+DMSO
Fer-1+iEV-NC
iEV-150+Fer-1
A375
A875
Absorbance at 490nm
0h
24h
48h
72h
D
C11-BODIPY
Relative Lipid Peroxidation
control
Erastin
iEV-150
iEV-150+Fer-1
E
MitoSOX
Relative Mitochondrial ROS
F
Relative Fe²+ Level
G
GSH/GSSG Value
H
A375
A875
Number
PI
G0/G1
S
G2/M
ns
Cell cycle distribution(%)
I
iEV-NC
iEV-150
Annexin V-FITC
FITC

Supplementary Figure 6 : iEV-150 induces ferroptosis and inhibits melanoma growth. **A** A875 cells co-cultured with iEV-150 were treated with RCD inhibitors—z-VAD-FMK (20 μM), Nec-1 (10 μM), Baf-A1 (70 nM), VX-765 (10 μM), or Fer-1 (5 μM)—and cell proliferation was assessed after 3 days. **B** A875 cell spheroid formation assay. **C** CCK-8 assay was used to evaluate the proliferation of melanoma cells treated in different groups. **D** The levels of lipid ROS were analyzed in A875 cells from the co-culture iEV-150 group, the Erastin group, and the group co-cultured with iEV-150 and treated with Fer-1. **E** The mitochondrial superoxide levels in A875 cells from each group were analyzed. **F–G** The Fe^{2+} levels and GSH/GSSG ratio were analyzed in A875 cells from each group. **H** Flow cytometry analysis of the cell cycle in different treatment groups. **I** Flow cytometry analysis of cell apoptosis in different treatment groups. Mean ± SD, *$p < 0.05$; **$p < 0.01$; ***$p < 0.001$; ns, not significant.

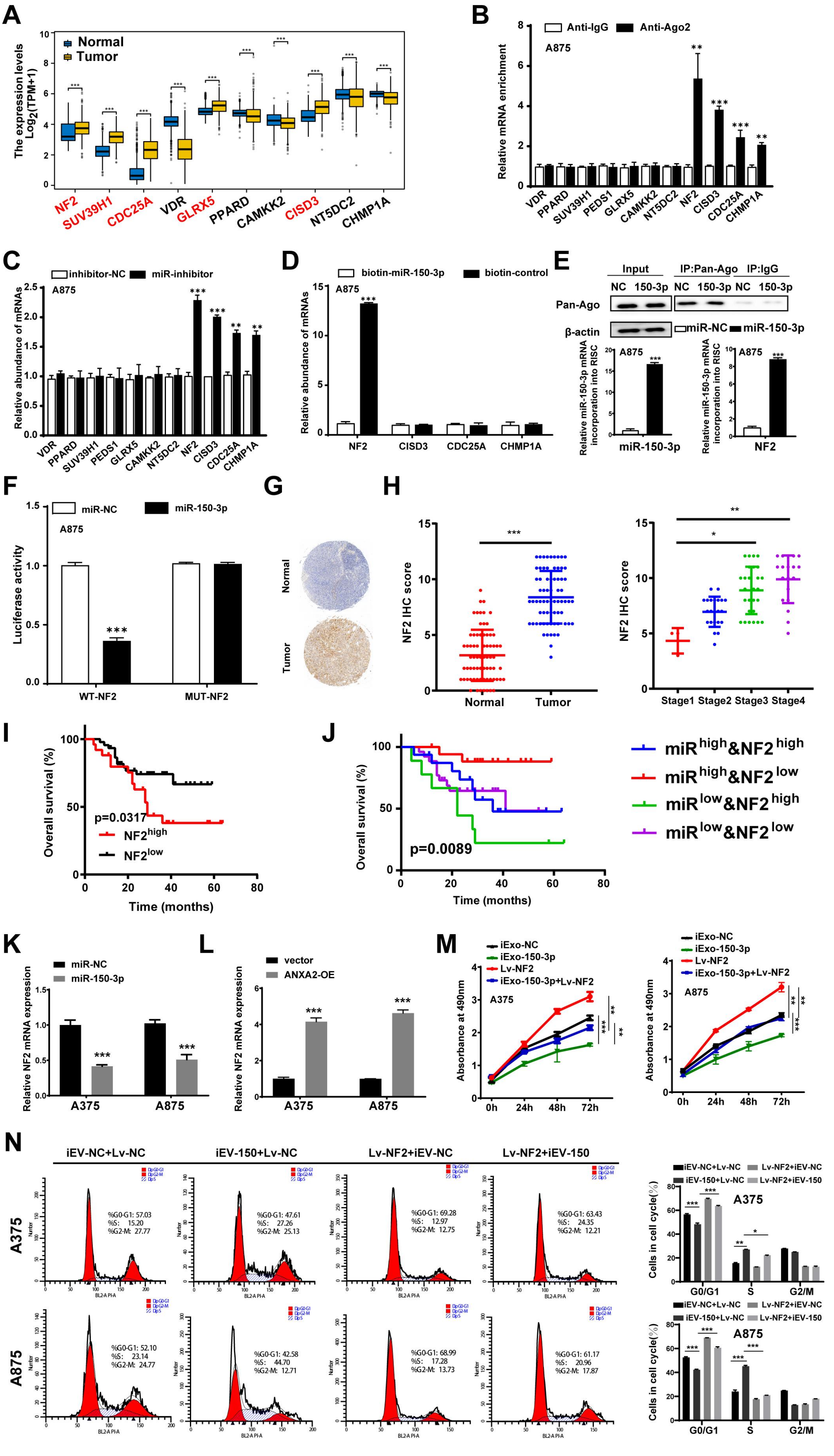

A
Normal
Tumor
The expression levels Log2(TPM+1)
NF2
SUV39H1
CDC25A
VDR
GLRX5
PPARD
CAMKK2
CISD3
NT5DC2
CHMP1A
B
Anti-IgG
Anti-Ago2
A875
Relative mRNA enrichment
VDR
PPARD
SUV39H1
PEDS1
GLRX5
CAMKK2
NT5DC2
NF2
CISD3
CDC25A
CHMP1A
C
inhibitor-NC
miR-inhibitor
A875
Relative abundance of mRNAs
D
biotin-miR-150-3p
biotin-control
A875
Relative abundance of mRNAs
NF2
CISD3
CDC25A
CHMP1A
E
Input
IP:Pan-Ago
IP:IgG
NC
150-3p
Pan-Ago
β-actin
miR-NC
miR-150-3p
A875
Relative miR-150-3p mRNA incorporation into RISC
miR-150-3p
NF2
F
miR-NC
miR-150-3p
A875
Luciferase activity
WT-NF2
MUT-NF2
G
Normal
Tumor
H
NF2 IHC score
Normal
Tumor
Stage1
Stage2
Stage3
Stage4
I
Overall survival (%)
p=0.0317
NF2high
NF2low
Time (months)
J
Overall survival (%)
p=0.0089
Time (months)
miRhigh&NF2high
miRhigh&NF2low
miRlow&NF2high
miRlow&NF2low
K
miR-NC
miR-150-3p
Relative NF2 mRNA expression
A375
A875
L
vector
ANXA2-OE
Relative NF2 mRNA expression
A375
A875
M
iExo-NC
iExo-150-3p
Lv-NF2
iExo-150-3p+Lv-NF2
A375
A875
Absorbance at 490nm
0h
24h
48h
72h
N
iEV-NC+Lv-NC
iEV-150+Lv-NC
Lv-NF2+iEV-NC
Lv-NF2+iEV-150
A375
A875
%G0-G1: 57.03
%S: 15.20
%G2-M: 27.77
%G0-G1: 47.61
%S: 27.26
%G2-M: 25.13
%G0-G1: 69.28
%S: 12.97
%G2-M: 12.75
%G0-G1: 63.43
%S: 24.35
%G2-M: 12.21
%G0-G1: 52.10
%S: 23.14
%G2-M: 24.77
%G0-G1: 42.58
%S: 44.70
%G2-M: 12.71
%G0-G1: 68.99
%S: 17.28
%G2-M: 13.73
%G0-G1: 61.17
%S: 20.96
%G2-M: 17.87
BL2-A PI-A
Number
iEV-NC+Lv-NC
iEV-150+Lv-NC
Lv-NF2+iEV-NC
Lv-NF2+iEV-150
Cells in cell cycle(%)
G0/G1
S
G2/M

Supplementary Figure 7： iEV-150 regulates melanoma cell proliferation by inhibiting NF2 expression. A Expression of candidate target genes analyzed in the TCGA-SKCM combined GTEx database. **B** AGO2-RIP-qRT-PCR analyzed mRNA enriched with miR-150-3p. **C** qRT-PCR to detect changes in candidate mRNA expression after inhibition of miR-150-3p. **D** miRNA pulldown assay. **E** In A875 cells overexpressing miR-150-3p, Pan-Ago2 antibody was used for immunoprecipitation (IP) of the Ago2/RISC complex, with IgG as a negative control and β-actin as an internal control. qRT-PCR analysis showed that, compared to the control, both miR-150-3p and NF2 were incorporated into the RISC complex in miR-150-3p-overexpressing A875 cells, using U6 and GAPDH as internal controls. **F** Luciferase reporter assay to evaluate the interaction between miR-150-3p and the 3'UTR of NF2. **G, H** The representative image (G) and data statistics (H) of NF2 expression in melanoma tissue microarray. **I, J** Based on the expression in the tissue microarray, the correlation between NF2 gene expression and survival time of melanoma patients (I) as well as the correlation between miR-150-3p combined with NF2 and survival time of melanoma patients (J) were analyzed. **K, L** qRT-PCR analysis of the change in NF2 expression after overexpressing miR-150-3p (K) and ANXA2 (L) separately. **M** CCK8 assay was used to detect the proliferation of melanoma cells under the conditions of iEV-150 co-culture, NF2 overexpression, or treatment with both. **N** Detection of melanoma cell cycle by flow cytometry. Mean ± SD, *p < 0.05; **p < 0.01; ***p < 0.001.

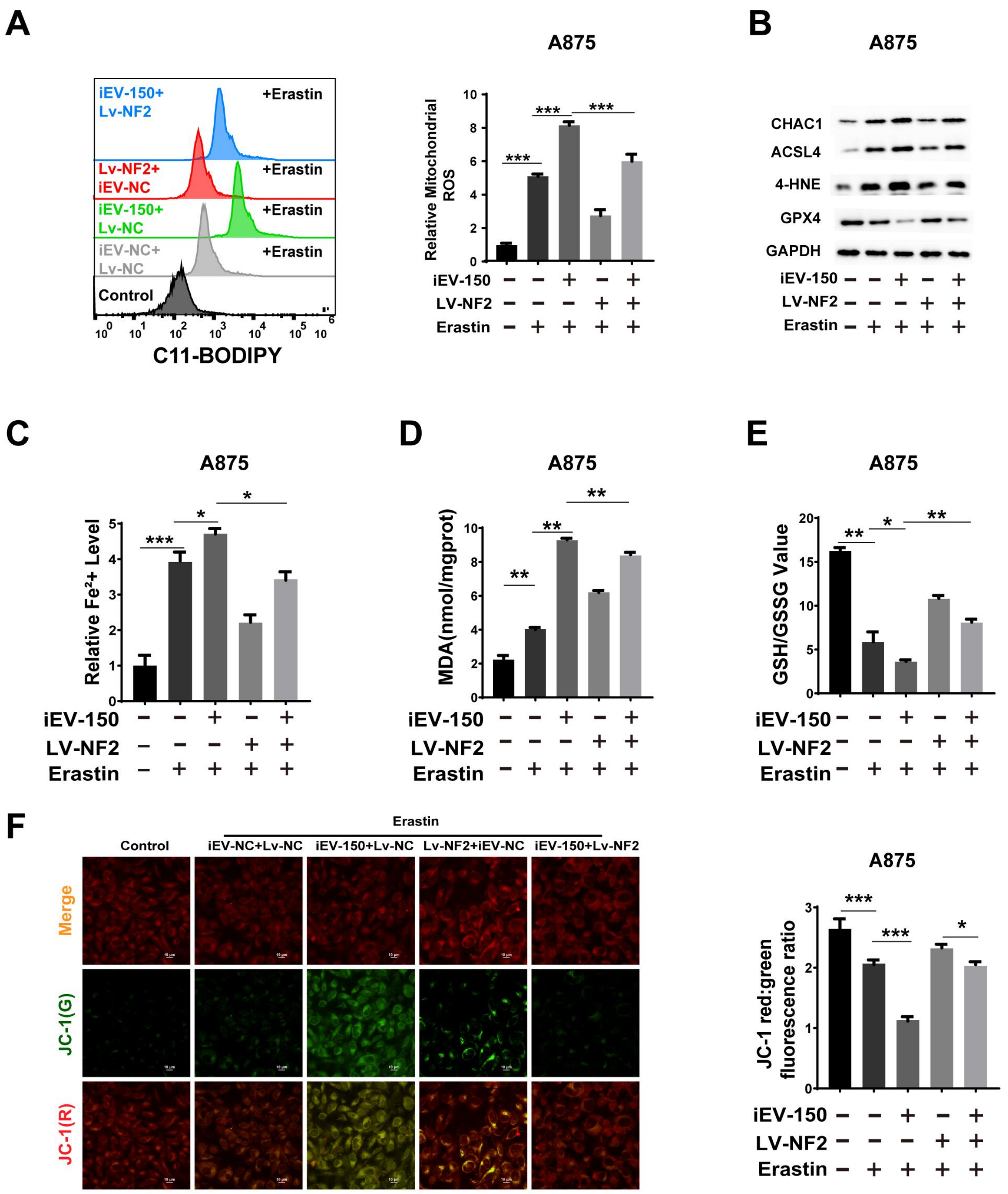

Supplementary Figure 8：iEV-150 promotes ferroptosis by reducing NF2 expression. A Flow cytometry analysis of lipid ROS levels after C11-BODIPY staining in cells. **B** Western blot detection of ferroptosis-related molecule expression in A385 cells after Erastin (15 μM) treatment combined with iEV-150 co-culture or NF2 overexpression, or treatment with all three. **C–F** Analysis of Fe^{2+} levels (C), MDA levels (D), GSH/GSSG ratio (E), mitochondrial membrane potential (F) under different treatment conditions in melanoma cells. Mean ± SD, $^{*}p < 0.05$; $^{**}p < 0.01$; $^{***}p < 0.001$.

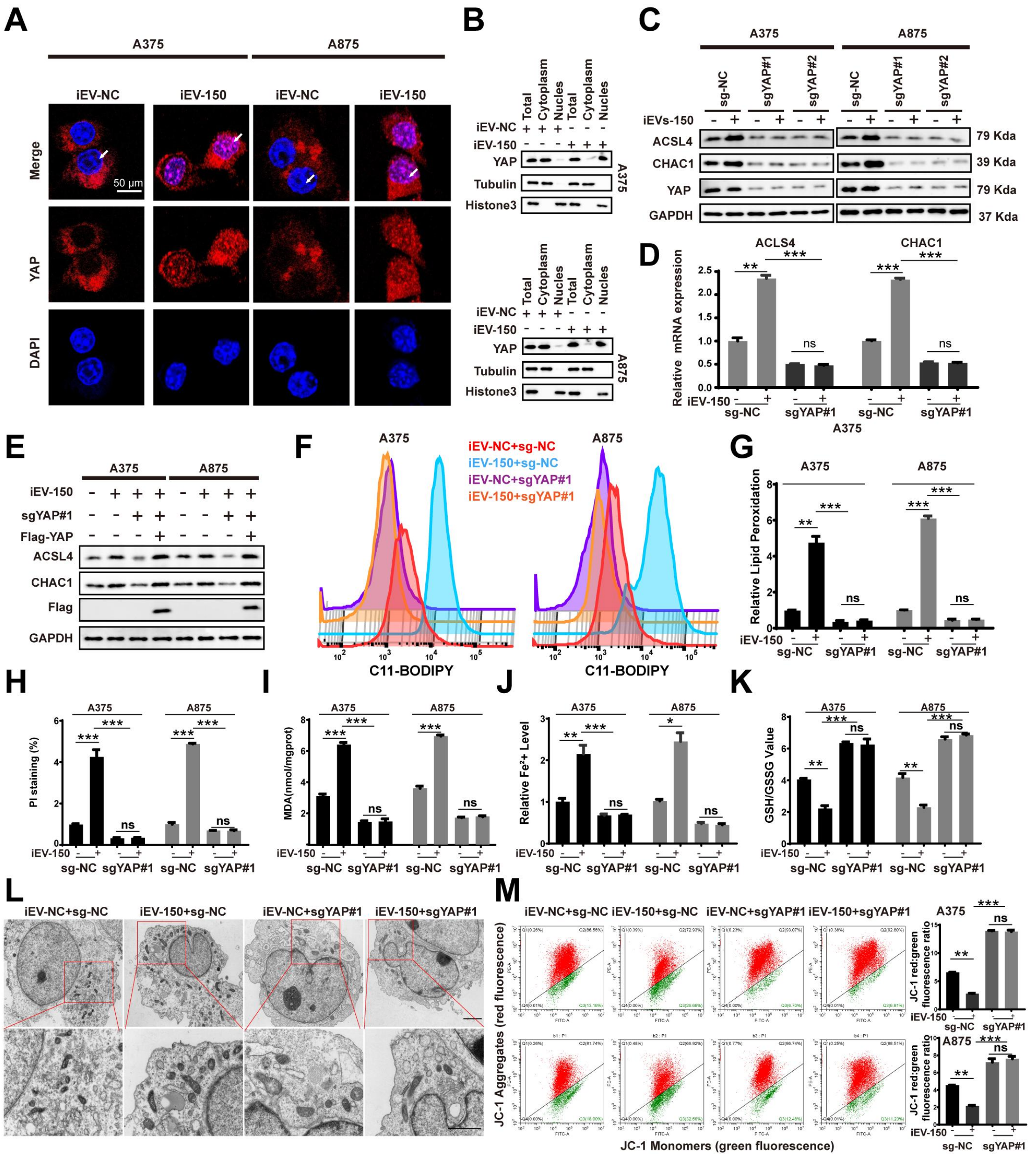

Supplementary Figure 9 iEV-150 regulates ferroptosis in a Hippo-YAP pathway-dependent manner. A Immunofluorescence detection of nuclear localization changes of YAP in melanoma cells after co-culture with iEV-150. **B** Western blot detection of YAP protein expression in the nucleus and cytoplasm of melanoma cells after co-culture with iEV-150. **C, D** iEV-150 promotes the expression of ACSL4 and CHAC1 in a YAP-dependent manner. The expression of ACSL4 and CHAC1 in control and YAP knockdown melanoma cells co-cultured with iEV-150 was detected by Western blot (C) and qRT-PCR (D). **E** Western blot detection of ACSL4 and CHAC1 expression in cells with YAP knockout and YAP overexpression after co-culture with iEV-150. **F–M** Assessed ferroptosis in control and YAP knockout melanoma cells co-cultured with iEV-150 by measuring lipid ROS levels (F, G), cell death percentage (H), MDA levels (I), iron ion levels (J), GSH/GSSG ratio (K), mitochondrial morphology (L), and mitochondrial MMP (M). Mean ± SD, *p < 0.05; **p < 0.01; ***p < 0.001; ns, not significant.

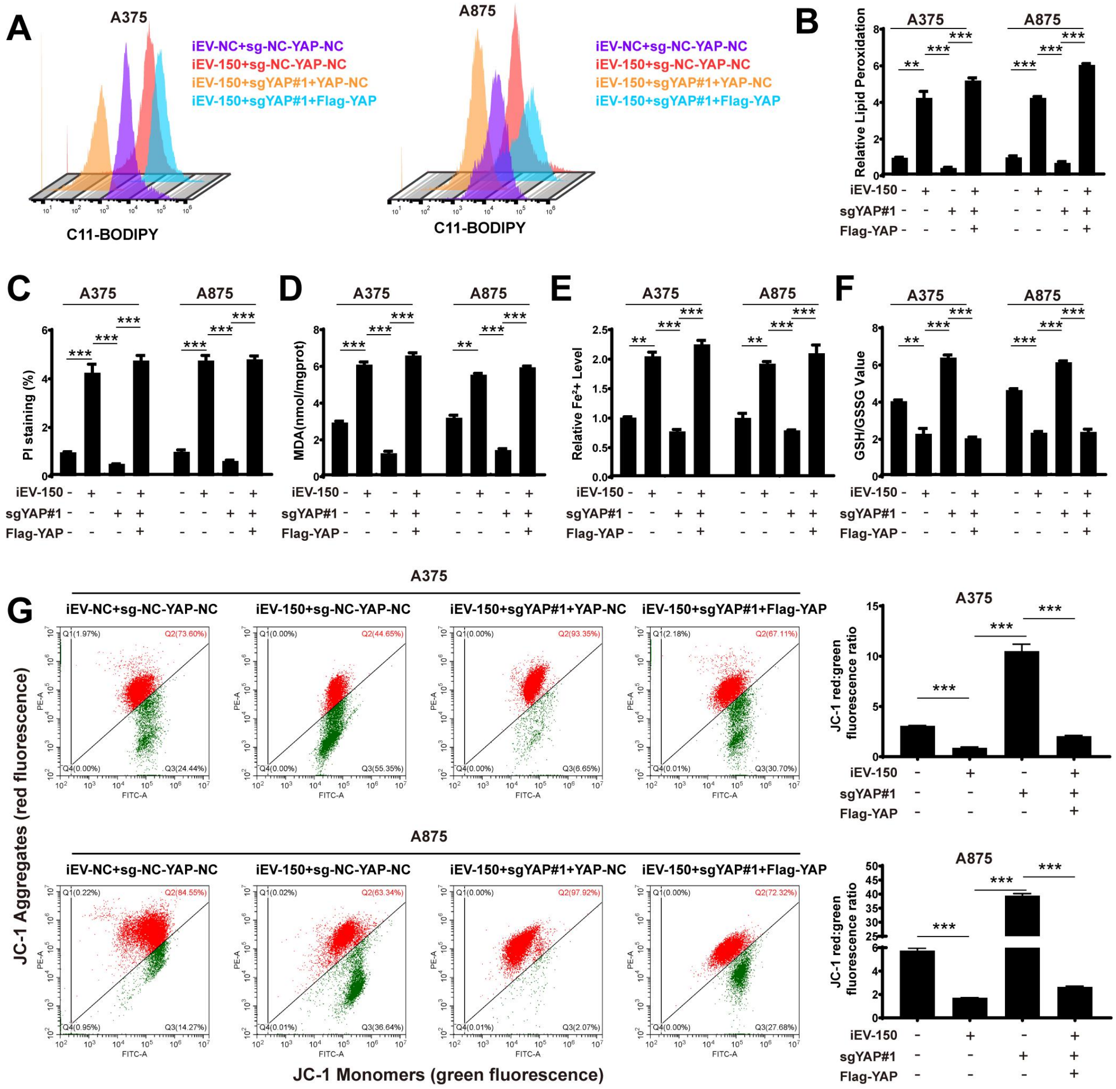

Supplementary Figure 10： iEV-150 regulates ferroptosis in melanoma cells by upregulating ACSL4 and CHAC1 through YAP. A, B Flow cytometry analysis of lipid ROS levels after C11-BODIPY staining in cells (A) and statistical analysis (B). **C–G** In YAP-rescued A375 and A875 cells, the effects on ferroptosis were evaluated by analyzing cell death percentage (C), MDA levels (D), ferrous ion levels (E), GSH/GSSG ratio (F), and mitochondrial membrane potential (G). Mean ± SD, *p < 0.05; **p < 0.01; ***p < 0.001.

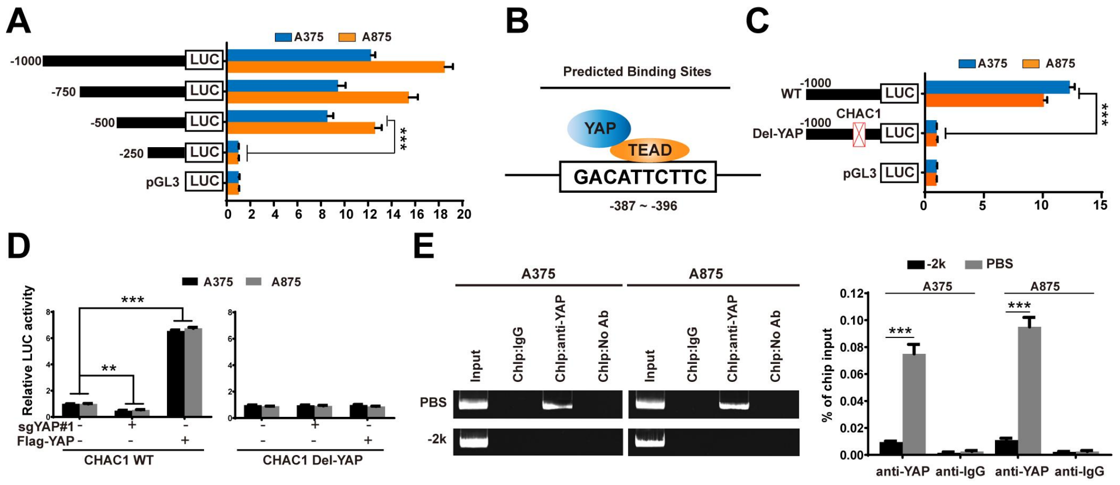

Supplementary Figure 11 YAP directly targets the CHAC1 promoter and promotes its transcription. A Measured luciferase activity using a dual-luciferase reporter system with a specific truncation of the CHAC1 promoter in A375 and A875 cells. **B** Predicted CHAC1 binding sites based on bioinformatics analysis (PBS). **C** deleted PBS, and the corresponding structure was designated as Del-YAP. Luciferase activity was measured in melanoma cells. **D** Detected CHAC1 promoter activity in A375 and A875 cells with simultaneous YAP knockout and overexpression using the dual-luciferase reporter system. **E** immunoprecipitated chromatin with anti-YAP antibody or negative control anti-IgG antibody, followed by qPCR in A375 and A875 cells. Mean ± SD, *p < 0.05; **p < 0.01; ***p < 0.001.

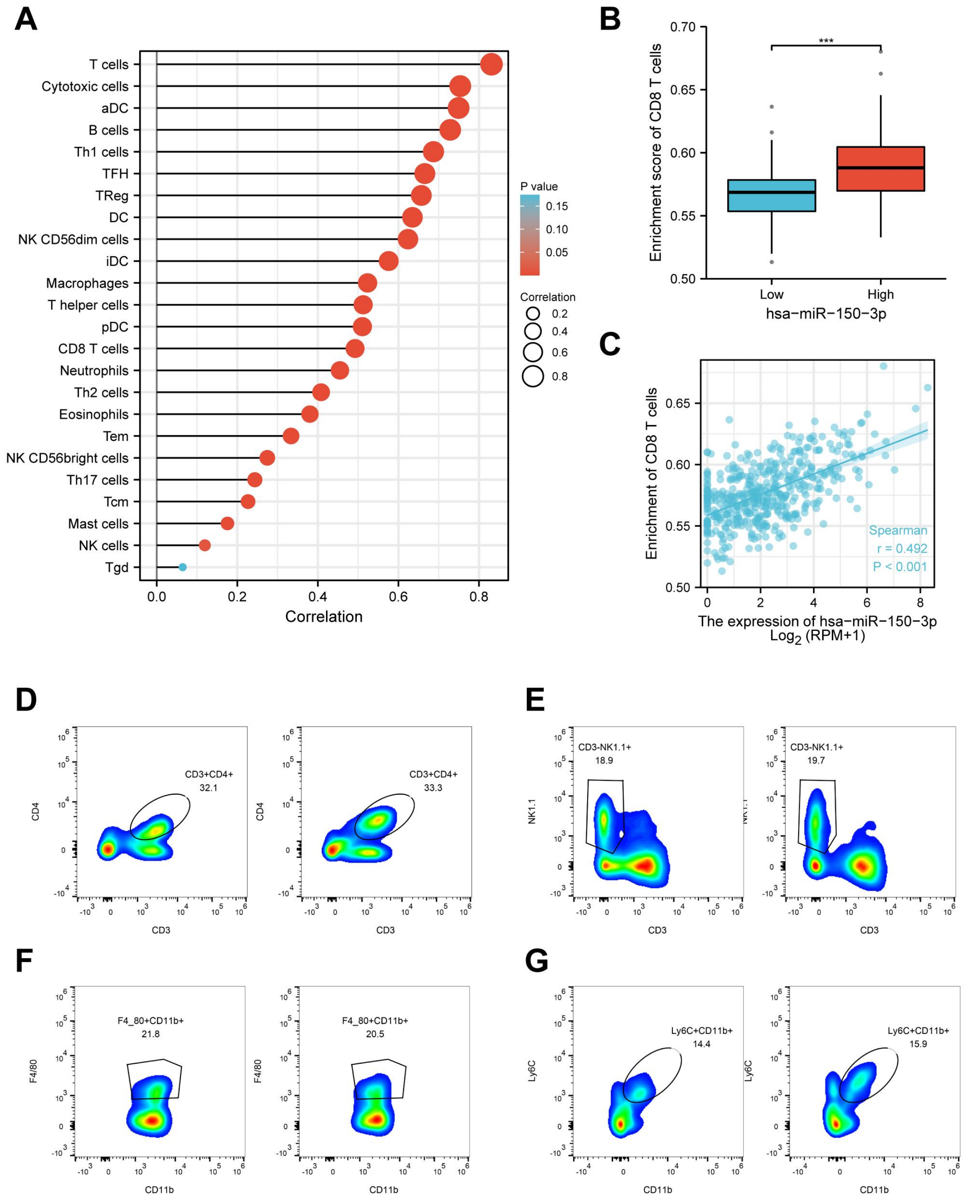

Supplementary Figure 12： iEV-150 is positively correlated with CD8+ T cells. A The correlation between miR-150-3p and tumor-infiltrating immune cells was evaluated by analyzing TCGA-SKCM data. **B** Bioinformatics analysis of CD8+ T cell enrichment in melanoma tissues with high and low expression of miR-150-3p. **C** Correlation analysis of miR-150-3p and CD8+ T cells in TCGA-SKCM data. **D–G** Flow cytometry analysis of the proportions of tumor-infiltrating CD4+ T cells (D), NK cells (E), dendritic cells (F), and macrophages (G) in mouse tumor tissues treated with iEV-150. Mean ± SD, *p < 0.05; **p < 0.01; ***p < 0.001.

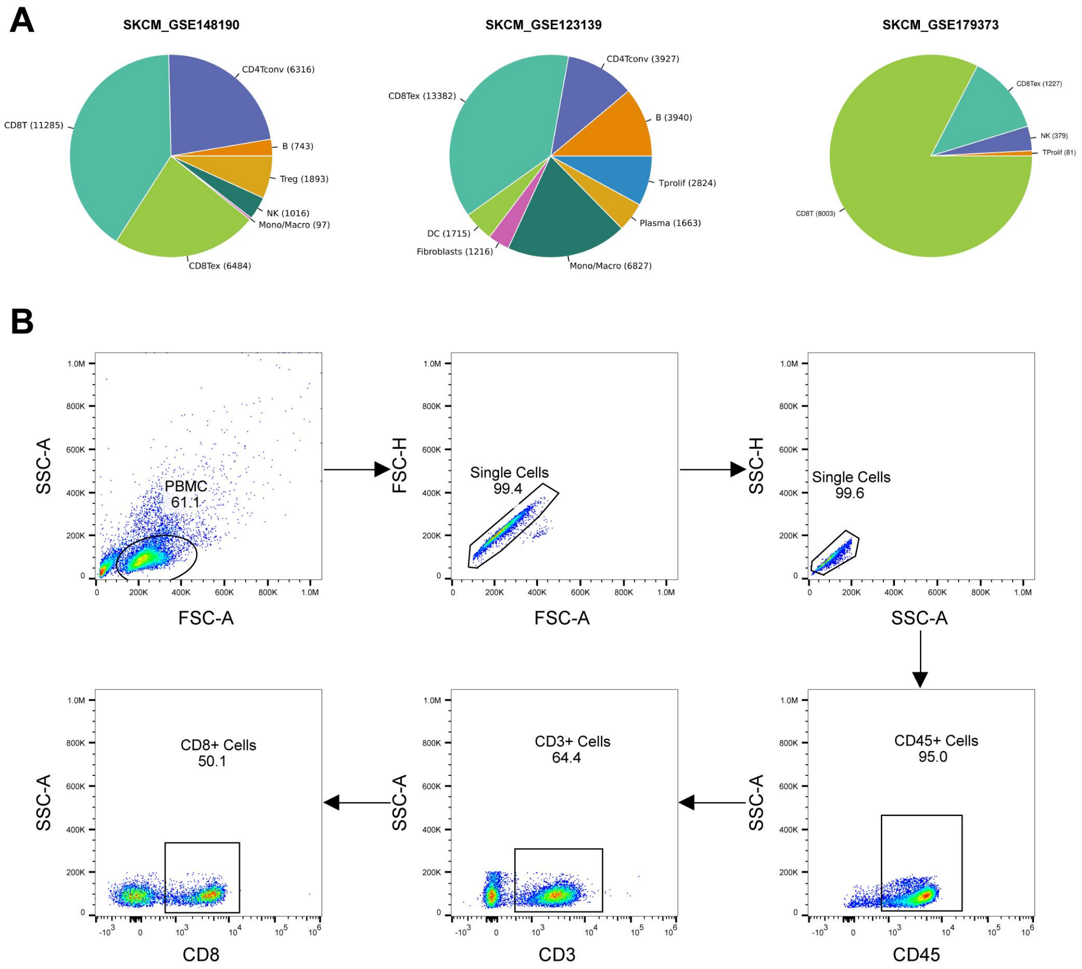

Supplementary Figure 13： Proportion of CD8+ T cells in melanoma patients and isolation from PBMC. A Pie chart showing the proportion of tumor-infiltrating immune cells in TCGA-SKCM tissues based on bioinformatics analysis. **B** CD8+ T cells were isolated from PBMCs by flow cytometry.

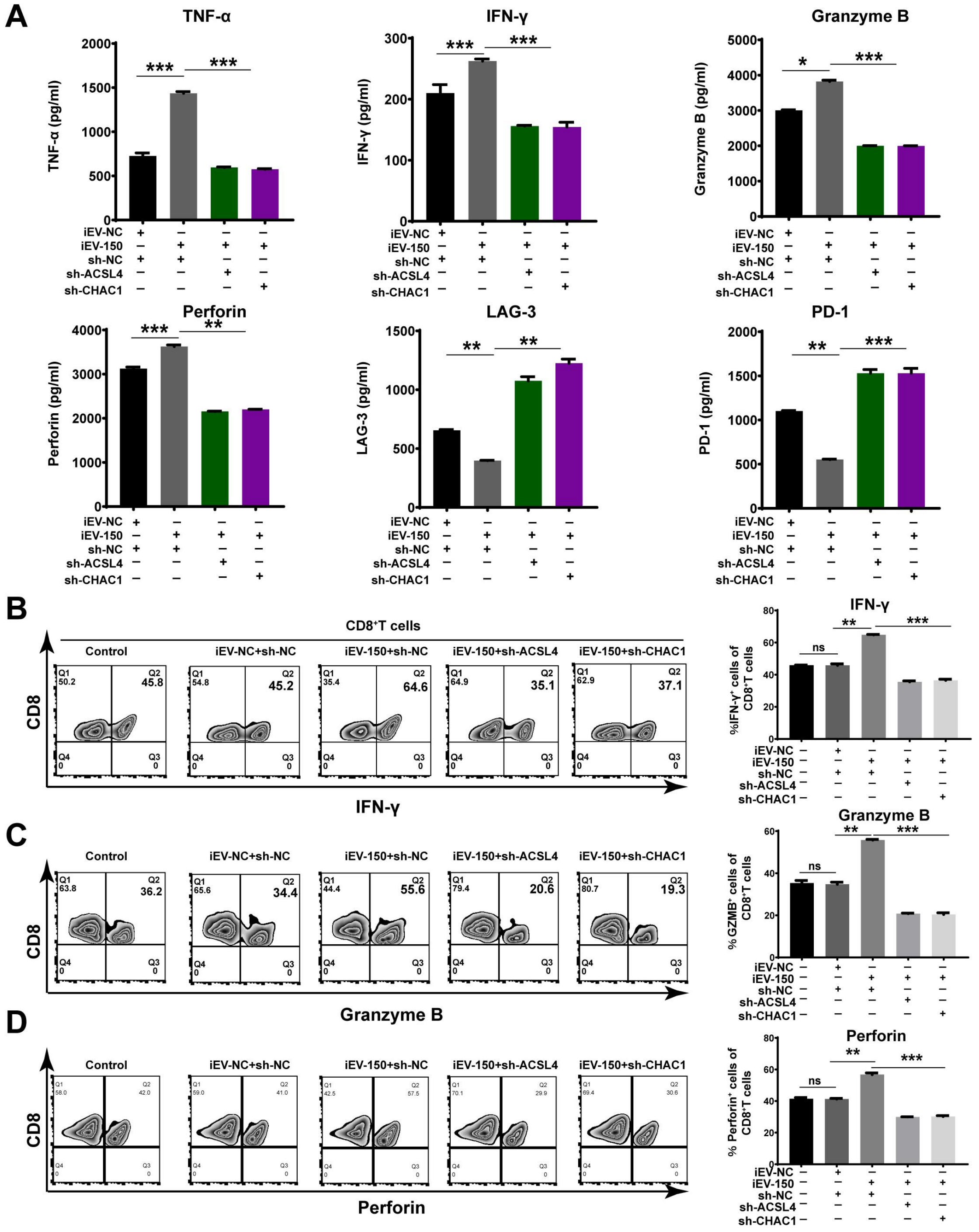

Supplementary Figure 14： iEV-150 regulates melanoma cells through the upregulation of ACSL4 and CHAC1, thereby modulating CD8+ T cells. A After co-culturing iEV-150 and sh-ACSL4/CHAC1 treated A375 cells with CD8+ T cells, TNF-α, IFN-γ, Granzyme B, Perforin, LAG-3, and PD-1 expression were detected using ELISA. **B–D** After co-culturing iEV-150 and sh-ACSL4/CHAC1 treated A375 cells with CD8+ T cells, IFN-γ, Granzyme B, and Perforin expression were detected by flow cytometry. Mean ± SD, $^{*}p < 0.05$; $^{**}p < 0.01$; $^{***}p < 0.001$.

Supplementary Table 1 miR-150-3p RNA binding protein candidates obtained from the mass spectrometric analysis

Prot_Name	Prot_Number	Prot_Score	prot_mass	Coverage (%)	Isoelectric Point	emPAI
ACTC	8	998	42334	34.7	5.23	6.63
ANXA2	31	463	38808	36.9	7.57	2.7
CKAP4	61	244	66097	20.9	5.63	0.55
ODB2	66	240	53852	15.8	8.71	0.92
TBA1B	69	236	50804	25.7	4.94	0.76
FLNB	71	233	280157	3.5	5.47	0.08
HNRH1	80	216	49484	8.7	5.89	0.38
PDIA6	91	192	48490	13.6	4.95	0.39
TFR1	92	189	85274	10.5	6.18	0.3
SVIL	93	188	249417	3.3	6.55	0.05
VDAC2	109	160	32060	14.3	7.49	0.48
AT1A1	110	159	114135	4.5	5.33	0.09
VAPB	120	148	27439	29.2	6.85	0.99
PGK1	127	142	44985	10.1	8.3	0.24
RLA0L	129	140	34514	13.9	5.41	0.32
ECHA	138	130	83688	5.4	9.16	0.12
HNRPF	140	129	45985	10.8	5.38	0.41
RL7A	146	127	30148	20.7	10.61	0.87
LDHB	155	117	36900	15.3	5.71	0.41
MERL	157	116	69874	9.6	6.11	0.32
FLOT1	159	116	47554	8.4	7.08	0.22
NB5R3	162	114	34441	9.6	7.18	0.32
H2A1	170	112	14083	20.8	10.9	1.38
FRG1	176	107	29439	15.9	9.11	0.38
HNRPK	177	107	51230	6.5	5.39	0.21
MIC60	180	105	84026	6.2	6.08	0.12
EIF3D	183	102	64560	7.5	5.79	0.16
ATD3B	184	102	73098	4.0	9.3	0.19
RL3	185	101	46365	9.2	10.19	0.23
MYO1B	186	100	132928	4.8	9.43	0.13
GGTL3	198	95	24201	5.8	5.75	0.14
GNAI3	201	94	41076	7.3	5.5	0.17
PCBP1	205	92	37987	6.7	6.66	0.18
PDIA4	206	91	73229	2.8	4.96	0.09
EF2	207	91	96246	4.0	6.41	0.11
RACK1	209	90	35511	5.0	7.6	0.2
SFXN1	210	90	35881	6.8	9.22	0.19
LDHA	211	90	36950	9.3	8.44	0.29
ESYT1	213	89	123293	2.3	5.57	0.05
GANAB	214	88	107263	3.2	5.74	0.06
CALX	219	86	67982	4.1	4.47	0.15

Prot_Name	Prot_Number	Prot_Score	prot_mass	Coverage (%)	Isoelectric Point	emPAI
ITB1	220	86	91664	3.3	5.27	0.07
AT2A2	224	84	116336	2.7	5.23	0.06
ROA2	225	84	37464	12.2	8.97	0.29
RB11B	229	82	24588	14.7	5.64	0.46
HLAB	230	82	40777	3.6	5.57	0.08
SRSF7	234	82	27578	16.8	11.83	0.41
CALR	236	81	48283	10.8	4.29	0.39
QCR2	237	80	48584	6.6	8.74	0.14
VAPA	238	80	28103	11.2	8.8	0.57
PPT1	239	79	34627	4.9	6.07	0.1
NMT1	242	78	57112	7.3	7.66	0.18
PODXL	243	78	59055	4.7	5.28	0.11
NOL6	246	77	128368	1.4	7.42	0.03
FUMH	247	77	54773	3.5	8.85	0.06
DDX3X	249	77	73597	3.9	6.73	0.09
SQOR	252	76	50214	3.1	9.18	0.07
FBRL	253	76	33877	9.3	10.18	0.21
RS24	255	75	15413	20.3	10.79	1.22
VAT1	256	74	42122	3.6	5.88	0.08
GT251	257	73	71933	9.0	6.85	0.2
GNL3L	259	73	66216	5.5	8.68	0.16
OAT	260	72	48846	5.7	6.57	0.14
DRG1	262	71	40802	7.4	9.0	0.17
HNRPQ	276	67	69788	2.1	8.68	0.05
BTF3	277	67	22211	16.0	9.41	0.52
MYOF	279	66	236100	1.1	5.84	0.03
M2OM	280	66	34211	5.1	9.92	0.1
MDHM	282	65	35937	7.7	8.92	0.19
ANXA1	283	65	38918	4.0	6.57	0.08
TM214	285	65	77957	1.7	9.28	0.04
RAC2	288	64	21814	17.2	7.52	0.77
LMNB1	289	64	66653	3.8	5.11	0.1
CAPON	290	63	56457	3.8	5.89	0.06
CCN1	291	63	44165	7.1	8.64	0.16
PDIA3	294	62	57146	11.5	5.98	0.32
MLEC	295	62	32385	4.5	5.27	0.1
CC124	297	61	25820	10.3	9.54	0.27
IMP3	298	61	21951	8.2	9.54	0.15
NCEH1	304	60	46064	2.9	6.76	0.07
RAB14	311	59	24110	12.1	5.85	0.3
ACSL3	312	59	81338	5.6	8.65	0.13
FLOT2	313	59	47434	2.8	5.19	0.07
GLYM	314	59	56414	5.8	8.76	0.12

Prot_Name	Prot_Number	Prot_Score	prot_mass	Coverage (%)	Isoelectric Point	emPAI
GBB4	319	57	38284	6.2	5.6	0.18
GBB1	320	57	38151	9.7	5.6	0.28
LC7L2	323	57	46942	7.4	10.02	0.15
SCRB2	325	56	54712	2.5	5.0	0.06
RL37A	326	56	10497	19.6	10.44	0.33
SRSF6	328	55	39677	5.2	11.42	0.17
TRAP1	330	55	80345	4.0	8.3	0.08
MIRO2	332	55	69101	3.1	5.55	0.1
RPN2	333	54	69355	3.6	5.44	0.05
RL15	334	54	24245	4.4	11.62	0.14
ENOA	336	53	47481	2.8	7.01	0.07
FXRD1	338	53	54120	5.1	7.66	0.13
FMNL3	339	52	118051	1.3	6.23	0.03
NDUS3	341	51	30337	9.8	6.99	0.23
TIMP3	342	51	24813	10.0	9.0	0.29
TM109	343	50	26194	4.9	10.48	0.13
OST48	345	50	50940	4.8	6.09	0.13
LAMP2	347	50	45503	2.0	5.35	0.07
IFRD1	348	50	51035	3.8	6.81	0.13
IF2B	349	50	38706	6.3	5.6	0.18
HBB	350	49	16102	15.6	6.75	0.47
SSRD	351	49	19158	6.4	5.76	0.18
PP1G	353	49	37701	4.6	6.13	0.09
VPP1	354	49	97148	1.3	6.02	0.03
LYSC	355	49	16982	8.1	9.38	0.2
PTCD3	356	49	79184	1.2	6.0	0.04
PLSL	357	49	70814	5.3	5.29	0.15
HNRH3	358	48	36960	5.2	6.37	0.19
TMX1	359	48	32170	4.3	4.92	0.1
VA0D1	360	48	40759	3.1	4.89	0.08
NDKA	366	46	17309	7.9	5.83	0.2
RLA2	367	46	11658	16.5	4.42	0.3
MAGB2	368	46	35426	3.4	8.87	0.09
GLU2B	369	46	60357	2.5	4.33	0.05
XAGE1	371	45	9300	33.3	9.65	0.89
GRSF1	372	45	53606	4.2	5.83	0.13
DDX50	373	45	83084	2.0	9.26	0.04
NDUS1	374	45	80443	6.2	5.89	0.13
SNP23	376	44	23682	6.6	4.89	0.14
SRSF3	377	44	19546	14.0	11.64	0.37
GTR1	378	44	54391	3.7	8.93	0.12
COIL	379	44	63254	1.7	9.2	0.05
ITA5	381	43	115605	1.3	5.5	0.03

Prot_Name	Prot_Number	Prot_Score	prot_mass	Coverage (%)	Isoelectric Point	emPAI
SFRP1	382	43	36274	3.2	9.1	0.09
RL7	383	43	29264	6.0	10.66	0.24
ECHM	384	43	31823	7.2	8.34	0.1
TMED9	385	43	27374	7.2	7.82	0.12
VATB2	386	43	56807	2.9	5.57	0.06
CAPZB	387	43	31616	3.6	5.36	0.11
ATP6	388	42	24801	4.4	10.09	0.14
RMXL3	389	42	115724	1.3	9.19	0.03
ARGL1	390	42	33197	4.8	10.35	0.1
RCN1	391	42	38866	4.8	4.86	0.09
H10	393	41	20850	7.7	10.84	0.35
PHLB2	395	41	142812	1.4	7.06	0.05
KDM2B	396	41	154969	0.9	8.85	0.02
BLMH	401	40	53155	2.4	5.87	0.06
RU2A	402	40	28512	3.5	8.72	0.12
RL27	403	40	15788	5.9	10.56	0.22
RBM39	406	39	59628	2.1	10.1	0.06
PRDX3	408	39	28017	4.3	7.67	0.12
DLDH	409	38	54713	2.2	7.95	0.06
LG3BP	411	38	66202	2.2	5.13	0.05
P4HA2	413	38	61263	1.9	5.49	0.05
RAB5C	415	38	23696	5.6	8.64	0.14
PIM1	417	38	36005	4.5	5.72	0.09
SP16H	418	38	120409	1.4	5.5	0.03
TRFM	419	37	81760	1.4	5.61	0.04
RL28	420	37	15795	8.0	12.02	0.22
KPYM	422	37	58470	4.0	7.96	0.12
TOIP1	424	37	66379	2.4	8.22	0.05
ARPC4	427	37	19768	6.5	8.53	0.17
SPTN1	429	37	285163	0.9	5.22	0.02
IRF9	434	36	44125	1.5	5.58	0.07
MMP14	439	35	66194	2.1	7.63	0.05
TMEDA	440	35	25131	4.1	6.97	0.13
BUD31	442	35	17559	6.9	9.1	0.19
KC1G1	443	35	48937	4.3	9.13	0.14
RL31	444	35	14454	11.2	10.54	0.24
ARP5L	445	35	16931	7.8	6.15	0.2
PPIA	446	35	18229	7.3	7.68	0.19
GDN	448	34	44202	3.0	9.35	0.07
EFHD2	449	34	26794	2.9	5.15	0.12
CD59	450	34	14795	9.4	6.02	0.23
RT34	451	34	25692	6.9	9.98	0.28
ETFB	453	34	28054	8.6	8.24	0.25

Prot_Name	Prot_Number	Prot_Score	prot_mass	Coverage (%)	Isoelectric Point	emPAI
STX7	455	33	29911	5.7	5.41	0.11
HMGB3	457	33	23137	6.5	8.48	0.15
LAS1L	458	33	83982	2.3	4.64	0.04
RAP1B	459	33	21040	6.0	5.65	0.16
CHD4	460	33	219407	0.7	5.62	0.03
CX7A2	461	33	9390	7.2	9.75	0.37
CALU	463	32	37198	2.5	4.47	0.09
SPB1	464	32	96898	0.9	8.53	0.03
LTOR1	465	32	17848	8.1	5.01	0.19
PELP1	466	32	120879	0.7	4.29	0.03
MGST1	467	32	17644	3.9	9.41	0.19
RBM3	468	32	17160	9.6	8.86	0.2
ICAM1	469	32	58587	2.6	8.31	0.06
DAD1	470	32	12660	10.6	6.52	0.27
AL3A2	471	32	55269	2.7	7.98	0.06
PYR1	472	32	245167	0.4	6.02	0.01
RPC22	473	32	14380	5.7	9.88	0.24
AT1B3	474	32	31834	5.4	8.58	0.1
FUT10	476	31	56629	1.3	8.62	0.06
NOL10	478	31	80822	2.3	8.64	0.08
IMB1	480	31	98420	1.4	4.68	0.03
TRPM5	481	31	132338	0.5	6.32	0.02
BOREA	482	31	31418	2.9	9.88	0.11
AK1A1	483	31	36892	3.1	6.32	0.09
XRN2	484	30	109426	1.3	7.26	0.03
BAG2	486	30	23928	5.2	6.25	0.14
S22A4	487	30	62514	2.0	6.85	0.11
WASC4	488	30	137343	0.4	7.1	0.02
IFRD2	489	30	55464	1.8	8.21	0.06
STT3B	492	30	94241	2.1	9.04	0.07
ANM5	493	30	73322	2.2	5.88	0.04
AAAT	495	30	57018	2.4	5.34	0.06
MGME1	497	29	39795	2.3	7.57	0.08
DHC24	498	29	60803	1.7	8.42	0.05
SNTG1	499	29	59130	2.1	6.24	0.06
RM33	501	29	7671	26.2	10.81	1.13
PWP1	502	29	56363	3.0	4.6	0.06
TACO1	503	29	32913	2.0	8.37	0.1
TRPV2	504	29	86838	1.3	5.56	0.04
PAN3	505	29	96293	0.7	8.8	0.03
TGM2	506	29	78420	1.0	5.11	0.04
NU153	507	29	155440	0.8	8.97	0.02
COPB2	508	29	103278	1.0	5.15	0.03

Prot_Name	Prot_Number	Prot_Score	prot_mass	Coverage (%)	Isoelectric Point	emPAI
CCD47	509	29	56123	2.1	4.76	0.06
EX3L2	510	29	46343	2.7	7.64	0.07
FXL14	511	29	46769	1.7	9.15	0.07
ASPH	513	28	86266	0.8	4.92	0.04
MCM5	514	28	83031	1.0	8.64	0.04
TBL3	515	28	90347	1.5	6.44	0.04
NSA2	516	28	30218	5.0	10.28	0.11
KPRP	518	28	67172	1.0	8.72	0.05
Z804B	520	28	155157	0.4	8.84	0.02
DNJB6	522	28	36122	3.4	9.17	0.09
ARF4	523	28	20612	3.9	6.59	0.16
SCND3	524	27	153452	0.4	6.29	0.02
BRAF	525	27	85125	0.8	7.29	0.04
KLHL6	526	27	71340	1.4	5.89	0.05
GRDN	527	27	216593	0.4	5.9	0.02
OGG1	528	27	39214	1.7	8.89	0.08
EIFCL	529	27	106091	1.5	5.45	0.03
ZMYM5	530	27	76139	2.2	8.5	0.04
ERH	531	27	12422	5.8	5.63	0.28
TTI2	533	27	57449	1.4	6.63	0.06
MRP3	534	27	170660	1.0	6.79	0.02
EDC4	537	27	152992	0.6	5.55	0.02
CD37L	539	26	39323	2.7	5.22	0.08
CDC5L	541	26	92422	1.0	8.22	0.04
STT3A	542	26	81104	2.4	8.28	0.08
LAT1	543	26	55659	3.6	7.9	0.06
HSPB1	544	26	22826	8.3	5.98	0.15
PAI1	546	26	45088	2.2	6.68	0.07
IGKC	547	26	11929	16.8	6.11	0.29
CC112	548	26	53589	2.0	9.48	0.06
NEBU	549	26	775393	0.2	9.11	0.0
ADAM8	554	26	91222	0.7	7.63	0.04
ACADV	555	26	70745	2.0	8.92	0.05
KIF5A	556	25	118161	0.6	5.65	0.03
UXS1	558	25	47661	1.9	8.99	0.07
RM38	559	25	44968	1.8	7.19	0.07
ATP5H	560	25	18537	5.6	5.21	0.18
SGPL1	561	25	64053	2.6	9.24	0.05
LGUL	562	25	20992	3.8	5.12	0.16
EHBP1	563	25	140559	0.9	5.24	0.02
CAND1	565	25	137999	0.7	5.52	0.02
UBX2A	566	25	29488	3.9	5.91	0.11
NIPA4	567	24	44432	1.7	7.06	0.07

Prot_Name	Prot_Number	Prot_Score	prot_mass	Coverage (%)	Isoelectric Point	emPAI
NOX5	568	24	87353	1.7	8.88	0.04
HS3S6	570	24	37505	2.6	10.8	0.09
TFB2M	571	24	45776	4.8	9.3	0.07
BAX	572	24	21285	6.8	5.08	0.16
VANG2	573	24	59905	1.7	9.27	0.05
NOP56	574	23	66408	1.7	9.24	0.05
CISY	575	23	51908	2.1	8.45	0.06
TDIF2	576	23	84817	1.7	5.86	0.04
MMEL1	577	23	89994	2.1	5.64	0.04
O2T27	579	23	36060	5.0	8.47	0.09
AHNK2	580	23	617383	0.2	5.2	0.01
NPAS2	582	23	92531	0.8	6.35	0.04
WNT5B	583	23	41665	2.5	8.8	0.08
E41L3	584	23	121458	0.6	5.09	0.03
RL18A	585	23	21034	5.7	10.73	0.16
TICRR	587	23	212721	0.5	9.0	0.02
ZN326	589	23	65955	2.7	5.08	0.05
AR6P4	590	23	26530	6.8	10.87	0.13
AMPE	591	23	109689	0.6	5.31	0.03
B3GT6	593	22	37513	4.3	9.72	0.09
RL21	594	22	18610	9.4	10.49	0.18
LS14A	595	22	50727	2.2	9.55	0.06
ACON	597	22	86113	1.9	7.36	0.04
NSDHL	598	21	42159	3.5	8.16	0.08
FKB10	599	21	64717	2.1	5.36	0.05
PACER	600	20	75121	1.2	5.69	0.04
ACD11	601	20	88007	0.9	8.23	0.04
KIF15	603	20	161030	0.4	5.75	0.02
LRCH4	605	20	74089	1.5	8.54	0.04
PRA11	606	19	56675	2.3	8.83	0.06
B4GT1	607	19	44291	2.0	8.88	0.07
BTK	609	18	76917	1.4	7.83	0.04
F205A	610	18	149713	0.9	8.62	0.02
SYSM	611	17	58702	1.5	8.35	0.06
ZN480	612	17	63322	1.1	9.26	0.05
ADAL	613	17	40467	4.5	5.89	0.08
RPF2	615	15	35731	2.3	10.0	0.09
VPS45	616	14	65435	2.3	8.41	0.05
PTAR1	617	14	46718	2.2	6.5	0.07
TCPZ	618	13	58444	1.3	6.23	0.06
LONP2	619	13	95070	1.2	6.88	0.03

The samples used for LC-MS analysis were derived from three independent biological

replicates, which were separately processed and pooled prior to mass spectrometry to increase input amount and detection sensitivity.

Supplementary Table 2 Data of sequences for PCR in this study

Gene	Sequences (5’-3’)
GAPDH	Forward: AGCACAATACCATTAAAAAGCCTCA<br>Reverse: ACTCGGGACTTGGCGCTCT
NF2	Forward: TGAGGATGAAGAAGATGTCCCA<br>Reverse: TGCAGTAATTTCCCAGCTGA
ANXA2	Forward: TGTGGAGACGCTGGGAAGAAG<br>Reverse: TGACCTCATCCACACCTTTGGT
RAB14	Forward: GCGAGTGCAAAAACGGGAG<br>Reverse: GAGGTCACTAGCAGCCACAG
TMED9	Forward: GGAGGCATGCTGAGAGTTCA<br>Reverse: CTGCTCCACTTGTTCCACCA
VAPA	Forward: GAGCCTGGCCTCGTCCTA<br>Reverse: CTGTGAAGGGGCCTTTGAAT
ARF4	Forward: GCTGCAGAAAATGCTTCTGGT<br>Reverse: GTGTTGCACAAGTGGCTTGA
VDR	Forward: GGCCGGACCAGAAGCCTTT<br>Reverse: CCACACACCCCACAGATCC
PPARD	Forward: CAGCCGGGACAGTGTTGTA<br>Reverse: TGGACCTCTACAGGGTGGTT
SUV39H1	Forward: CGCCTGAGAAATGACAGACT<br>Reverse: AGGTAATATTCCTGTTCGCGG
PEDS1	Forward: TACTCGGTGTTGTTGCAGGG<br>Reverse: GAAAGCCTTCCCCACAATGG
GLRX5	Forward: GGAGCTCCGACAAGGCATTA<br>Reverse: CCCCCTACAAACTCGCCATT
CAMKK2	Forward: CCCTTTTGGCTGTTTGGCAG<br>Reverse: AGCGGAGCCACTTTGCTG
NT5DC2	Forward: TGGAGCGCATGCAGACCTA

Gene	Sequences (5′-3′)
	Reverse: GGGAGGCCATGTAGAGGTCA
CISD3	Forward: AAGACCCCCATCAAGGTGGA
	Reverse: CGTCACAGAAGGGCTGCTT
CDC25A	Forward: AATACATTCCCTACCTCAGAAGC
	Reverse: GGCAGCCACGAGATACAGG
CHMP1A	Forward: TGACTATGAAGGGGGTGACCA
	Reverse: TGCTCGAACCTGTCCATCAC
CHAC1	Forward: GAAGATCATGAGGGCTGCAC
	Reverse: TGGTATCGTAGCCACCAAGC
ACSL4	Forward: ATACCTGGACTGGGACCGAA
	Reverse: CCAATCCTGCAGCCATAGGT
hsa-miR-150-3p	CTGGTACAGGCCTGGGGGACAG
hsa-miR-1246	AATGGATTTTTGGAGCAGG
hsa-miR-191-5p	CAACGGAATCCCAAAAGCAGCTG

Supplementary Table 3 Antibodies in this studies

Reagent or Resource	Source	Cat no.
Antibodies		
anti-CD81	Abcam	Cat#ab109201
anti-TSG101	Abcam	Cat# ab125011
anti-CD63	Abcam	Cat#ab134045
anti-CD9	Abcam	Cat#ab263019
anti-GPX4	CST	Cat#52455
anti-HSP70	Abcam	Cat# ab181606
anti-4-HNE	Abcam	Cat#ab46545
anti-N-Cadherin	Proteintech	Cat#22018-1-AP
anti-Calnexin	Abcam	Cat#ab133615
anti-ANXA2	Abcam	Cat#ab154113
anti-E-Cadherin	Proteintech	Cat#20874-1-AP
anti-PCNA	CST	Cat#13110
anti-CCNE1	CST	Cat#20808
anti-Ki67	Proteintech	Cat#27309-1-AP
anti-CDK2	Proteintech	Cat#83635-4-RR
anti-P21	Proteintech	Cat#10355-1-AP
anti-CD31	Abcam	Cat#ab28364
anti-Vimentin	Proteintech	Cat#10366-1-AP

Reagent or Resource	Source	Cat no.
anti-BRCA1	Proteintech	Cat#22362-1-AP
anti-γH2AX	Abcam	Cat#ab2893
anti-RAD51	Proteintech	Cat#14961-1-AP
anti-β-actin	Abcam	Cat#ab8227
anti-GAPDH	ABclonal	Cat#AC001
anti-PALB2	Proteintech	Cat#14340-1-AP
anti-CHAC1	Proteintech	Cat#15207-1-AP
anti-ACSL4	Santa Cruz	Cat#sc-365230
anti-GPX4	Proteintech	Cat#67763-1-Ig
anti-NF2	Proteintech	Cat#ab84550
anti-GST	ABclonal	Cat#AE077
anti-Flag	CST	Cat#14793
anti-HA	CST	Cat#5017
anti-His	CST	Cat#12698
anti-p-LATS1(S909)	CST	Cat#9157
anti-p-LATS1(T1079)	CST	Cat#D57D3
anti-LATS1	CST	Cat#9153S
anti-YAP	CST	Cat#4912
anti-p-YAP(S127)	CST	Cat#4911S
anti-Histone3	Proteintech	Cat#68345-1-Ig
anti-CD8	Abcam	Cat#ab4055
anti-CD3	Abcam	Cat#ab16669
anti-cleved caspase3	Abcam	Cat#ab2302
anti-GSDMD-N	Abcam	Cat#ab215203
Mouse IgG	Immunoway	Cat#RS0001
Rabbit IgG	Immunoway	Cat#RS0002
alpha V beta 3	Abcam	Cat#Ab190147
alpha V+beta 5	Abcam	Cat#Ab177004

Supplementary Table 4 Data of sequences for shRNA and sgRNA in this study

Gene	Sequences (5’-3’)
sh-NC	TTCTCCGAACGTGTCACGT
sh-ACSL4	CCAGTGTTGAACTTCTGGAAA
sh-CHAC1	ACAACCTTGAATACTTGCT
sg-NC	GTGTAGTTCGACCATTCGTG
sg-YAP#1	GTGCACGATCTGATGCCCGG
sg-YAP#2	TCGAACATGCTGTGGAGTCA

Gene	Sequences (5′-3’)
sg-ANXA2#1	GGATCAGCATCATGACCGAG
sg-ANXA2#2	GGTCCTTCTCTGGTAGGCGA
